# Newly identified properties of known pharmaceuticals and myxobacterial small molecules revealed by screening for autophagy modulators

**DOI:** 10.1111/febs.70243

**Published:** 2025-08-28

**Authors:** Janine Fichtner, Yan Yan Beer, H. G. Mauricio Ramm, Sabrina Mühlen, Frank Surup, Jennifer Herrmann, Toni Luise Meister, Stephanie Pfaender, Ursula Bilitewski, Mark Brönstrup, Rolf Müller, Manfred Wirth, Eeva‐Liisa Eskelinen, Ingo Schmitz

**Affiliations:** ^1^ Systems‐Oriented Immunology and Inflammation Research Group Helmholtz Centre for Infection Research Braunschweig Germany; ^2^ Institute of Biomedicine University of Turku Finland; ^3^ Department of Molecular Immunology Ruhr University Bochum Germany; ^4^ Department of Microbial Drugs Helmholtz Centre for Infection Research Braunschweig Germany; ^5^ German Centre for Infection Research (DZIF), Partner site Hannover‐Braunschweig Germany; ^6^ Department of Pharmacy Helmholtz Institute for Pharmaceutical Research Saarland (HIPS), Helmholtz Centre for Infection Research (HZI) and Saarland University Saarbrücken Germany; ^7^ Department of Molecular and Medical Virology Ruhr University Bochum Germany; ^8^ Institute for Infection Research and Vaccine Development (IIRVD), Centre for Internal Medicine University Medical Centre Hamburg‐Eppendorf (UKE) Germany; ^9^ Department for Clinical Immunology of Infectious Diseases Bernhard Nocht Institute for Tropical Medicine, Hamburg (BNITM) Germany; ^10^ German Centre for Infection Research (DZIF), Partner site Hamburg‐Lübeck‐Borstel‐Riems Germany; ^11^ Research Unit Emerging Viruses Leibniz Institute of Virology Hamburg Germany; ^12^ Institute of Virology and Cell Biology University of Lübeck Germany; ^13^ Working Group Compound Profiling and Screening Helmholtz Centre for Infection Research Braunschweig Germany; ^14^ Department of Chemical Biology Helmholtz Centre for Infection Research Braunschweig Germany

**Keywords:** anti‐infectives, ATG4D, autophagy, drug repurposing, drug screening, LC3, natural products

## Abstract

Autophagy is a cellular degradation and recycling process important for maintaining cellular health and function. It is constitutively active at a low level in eukaryotic cells and can be induced by conditions of cellular stress, such as nutrient starvation. Moreover, autophagy plays an important role in diverse processes such as immunobiology, pathogen infection, ageing, and neurodegenerative and other diseases. Using a high‐content fluorescence assay for microtubule‐associated protein 1 light chain 3 beta (LC3B), a major player in the autophagic pathway, we screened a library of commercial drugs and natural products for activators and inhibitors of LC3B‐positive vesicle accumulation. Positive hits for known autophagy modulators included anisomycin, amphotericin B, carbonyl cyanide m‐chlorophenylhydrazone (CCCP) and cytochalasin D. Importantly, we identified several new autophagy modulators, such as aciclovir and myxobacterial vioprolides. Anisomycin, aciclovir and vioprolides promoted intracellular growth of *Staphylococcus aureus*, a bacterium that is known to be a target of autophagy. In contrast, anisomycin strongly inhibited influenza A virus and SARS‐CoV‐2 replication. Subsequently, we investigated the influence of these autophagy modulators in a cellular disease model of neuronal vacuolation and spinocerebellar degeneration (NVSD), which is associated with cysteine protease ATG4D mutations. We provide evidence that anisomycin and famciclovir, an aciclovir analogue, can normalise the elevated amount of LC3‐positive vesicles in mutant fibroblasts, highlighting their potential for the treatment of NVSD. Thus, the screening method enabled the identification of autophagy‐modulating compounds with therapeutic potential.

## Graphical abstract

Autophagy is a cellular degradation process involved in, for example, immune responses to pathogens and neurodegeneration. To identify modulators of autophagy, we developed a microscopy‐based screening assay and identified previously unknown autophagy‐modulating activities in known drugs and natural products from myxobacteria and fungi. Our data point to potential applications in infections with respiratory viruses and neuronal vacuolation and spinocerebellar degeneration (NVSD).

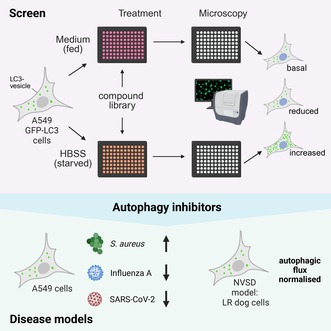

**Abbreviations**
ATG: autophagy‐related
CASM: conjugation of ATG8 to single membranes
CCCP: carbonyl cyanide m‐chlorophenylhydrazone
GFP: green fluorescent protein
IAV: Influenza A virus
LAMP2: lysosome‐associated membrane protein 2
LC3: microtubule‐associated protein 1A/1B‐light chain 3
LDELS: LC3‐dependent extracellular vesicle loading and secretion
LR: Lagotto Romagnolo
MAPK: mitogen‐activated protein kinases
MDCK: Madin‐Darby canine kidney
MOI: multiplicity of infection
mTOR: mammalian target of rapamycin
NOP14: Nucleolar Protein 14
NVSD: vacuolation and spinocerebellar degeneration
SQSTM1: Sequestosome 1 (aka p62)
TCID50: 50% tissue culture infectious dose

## Introduction

Macroautophagy, also referred to as autophagy, is initiated by the formation of a membrane structure known as an isolation membrane or phagophore that elongates to surround a portion of the cytoplasm [1, 2]. The fusion of the open ends of the phagophore results in a double‐membraned structure known as the autophagosome [1, 2]. The outer membrane of the autophagosome then fuses with a lysosome to form an autolysosome in which the inner autophagosomal membrane, together with the sequestered material, is degraded by lysosomal hydrolases. The resulting macromolecules are recycled to the cytosol for reuse, for example, for protein synthesis or energy production [2, 3, 4].

Autophagy is mediated by autophagy‐related (ATG) proteins that are highly conserved among eukaryotic species [2]. At the core of the autophagic machinery are two ubiquitin‐like conjugation systems required for efficient autophagosome biogenesis. First, the ubiquitin‐like molecule ATG12 is conjugated to ATG5 [5]. Together with ATG16L1, the ATG12‐ATG5 complex associates with nascent phagophores and acts as an E3‐like ligase to conjugate the ubiquitin‐like molecule ATG8 to the lipid phosphatidylethanolamine [6]. While only one ATG8 protein exists in yeast, mammals have evolved six homologues, that is, LC3A, LC3B, LC3C, GABARAP, GABARAPL1 and GATE‐16/GABARAPL2 [5]. ATG8 proteins are recruited to associate with phagophores after lipidation. Vesicular localisation of endogenous LC3 isoforms or a stably expressed GFP‐LC3B fusion construct is commonly used as a marker for autophagosomes [7]. Lipidated LC3 at the cytoplasmic surface of autophagosomes can be recycled by ATG4‐mediated cleavage. The mammalian ATG4 has four isoforms A, B, C and D. These cysteine proteases both prime LC3 for lipidation by cleaving the C terminus and deconjugate LC3 from its lipid modification to release it back to the cytosol [8]. Of the four mammalian ATG4 isoforms, ATG4D is the main ATG8‐delipidating enzyme in cells [9].

Autophagy is induced by various cellular stresses, such as nutrient starvation or cytotoxic harm, to prevent cell damage and promote survival [2, 10]. Thus, autophagy primarily has cytoprotective functions and is important for adaptation to different environmental conditions, for intracellular quality control of organelles and large protein complexes, as well as for normal developmental processes. Defects in autophagy are associated with various human diseases, including cancer and neurodegenerative diseases [1, 11]. For instance, ATG4D is protective against cerebellar neurodegeneration, and mutations in ATG4D are associated with a neurodevelopmental disorder in humans [9, 12]. Similarly, neurodegenerative vacuolar storage disease (NVSD) is a neurodegenerative disease of Lagotto Romagnolo dogs caused by a point mutation in ATG4D [13, 14]. Therefore, pharmacological modulation of autophagy is of great interest and has considerable potential for the development of novel therapeutics.

Another cellular stress is infection and, thus, selective autophagy, also known as xenophagy, has been shown to act as an antimicrobial mechanism to fight bacteria and viruses [15, 16, 17]. However, many pathogens have evolved mechanisms to subvert or exploit autophagy for their benefit [15, 16, 17]. For instance, we showed that the Gram‐positive bacterium *Staphylococcus aureus* inhibits its degradation by activating the host kinase p38 and thereby inhibiting the fusion of *S. aureus*‐containing autophagosomes with lysosomes [18]. On the contrary, it was recently shown that coronaviruses, including SARS‐CoV‐2, employ autophagy‐related genes as host factors for replication [19]. Therefore, pharmacological modulation of autophagy might be useful for the treatment of infectious diseases.

To facilitate the identification of autophagy‐modulating compounds, we developed a robust and sensitive high‐content fluorescence microscopy assay based on the human lung cancer cell line A549 stably expressing a GFP‐LC3 fusion protein. The A549 GFP‐LC3 cell line was employed to measure differences in the amount of GFP‐LC3‐positive vesicles under basal and starvation conditions using an automatic fluorescence microscope. Changes in the amount of GFP‐LC3‐positive vesicles were analysed by determining their number, size and intensity. The system was validated with known autophagy modulators and subsequently utilised to screen a library of commercially available substances as identifying novel applications for known drugs, a process called repurposing, is an attractive approach to reduce costs and time in drug discovery and development [20]. Additionally, a natural product library containing unique secondary metabolites derived from fungi and myxobacteria was screened, and several compounds that influenced autophagy were identified. Because autophagy was found to impact the proliferation of intracellular bacteria and viruses [15, 21], we further characterised hit compounds in assays probing for the replication of intracellular *S. aureus* as well as that of the respiratory viruses influenza A and SARS‐CoV‐2. While intracellular *S. aureus* survived better upon autophagy inhibition, replication of the two respiratory viruses was efficiently blocked by the identified autophagy inhibitors. Furthermore, we investigated the effects of these compounds on autophagy in NVSD fibroblasts because this cellular system offers insights into their capacity to modulate basal autophagy. Here, anisomycin and famciclovir normalised the amount of basal LC3‐positive vesicles in mutant NVSD fibroblasts. In summary, our screening approach identified several novel autophagy modulators, and our data imply that inhibition of basal autophagy is a potential therapeutic option for the treatment of SARS‐CoV‐2 infection and NVSD.

## Results

### Establishing an autophagy assay for compound screening

**Fig. 1:**
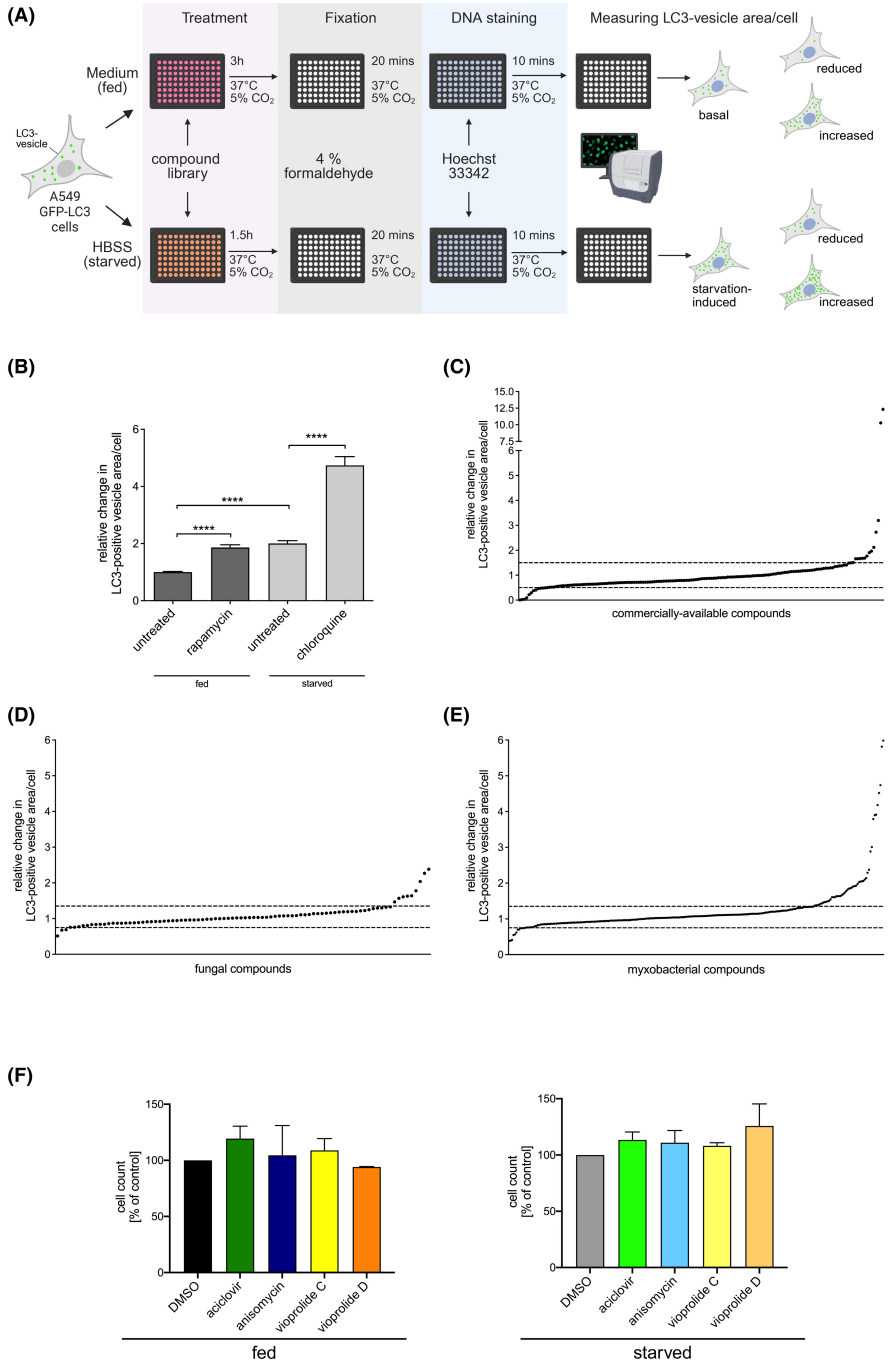
Establishment of an autophagy assay for compound screening and identification of chemical and natural compounds modulating autophagy. (A) Screening procedure. Amino acid starvation was induced by culturing cells in Hank's buffered salt solution (HBSS). Created in BioRender. Schmitz, I. (2025) https://BioRender.com/qk88jpv (B) Relative changes in the GFP‐LC3‐positive vesicle area per cell caused by the selected control treatments (1 μm rapamycin in full culture medium for 3 h, serum and amino acid starvation without and with 60 μm chloroquine for 1.5 h) compared with the negative control (fed and not treated). Results are from 16 independent experiments. Each bar represents the mean and standard deviation from 32 samples. Statistical indication according to unpaired two‐sided *t*‐test: ****P < 0.0001. (C) Relative change in the GFP‐LC3‐positive vesicle area per cell caused by known bioactive and commercially available compounds compared with the negative control (see Materials and methods section for details). Compounds were sorted according to their ability to modulate the level of GFP‐LC3‐positive vesicles. All compounds that increased or decreased the amount of GFP‐LC3‐positive vesicles more than a range of three standard deviations around the negative control (dotted lines) are classified as hits. Results are from two independent experiments. (D) Relative change in the GFP‐LC3‐positive vesicle area per cell caused by myxobacterial compounds compared with the negative control. Analysis was done as in (C). Results are from two independent experiments. (E) Relative change in the GFP‐LC3‐positive vesicle area per A549 cell caused by fungal compounds compared with the negative control. Analysis was done as in (C). Results are from two independent experiments. (F) Cell viability was calculated by determining the number of Hoechst 33342‐stained nuclei relative to the dimethyl sulfoxide (DMSO) solvent control in fed (left panel) and starved cells (right panel). The compounds were used at 10 μm for 3 (fed) or 1.5 h (starved). Results are from two experiments done in duplicate. Bar graphs represent the mean; error bars represent SEM.

To identify novel autophagy‐modulating compounds, we developed a robust and sensitive high‐content fluorescence microscopy assay employing the human lung cancer cell line A549 stably expressing a GFP‐LC3 fusion construct (Fig. 1A). Changes in the number, size and intensity of GFP‐LC3‐positive vesicles were analysed. First, we aimed to validate the robustness of the assay. A549 GFP‐LC3 cells were seeded in 96‐well plates, and autophagy was induced or blocked to evaluate whether the assay could discriminate between these treatments. Among the control treatments was rapamycin [22], which is widely used as an activator of autophagy due to its inhibitory effect on the mTOR kinase [23, 24]. Starvation by incubating the cells in serum‐ and amino acid‐free medium was also used to activate autophagy [23, 25]. To block autophagy at a late stage, chloroquine, a potent inhibitor of autophagosome‐lysosome fusion and widely used antimalarial drug [26], was employed under starvation conditions in order to accumulate autophagosomes, which occurs when a late‐stage autophagy inhibitor blocks their clearance. A reduction in the calculated vesicle area per cell suggests potential inhibitory effects of a compound on early steps in the autophagic process, that is, autophagosome formation, or a potential stimulatory effect on autophagosome clearance. An increase in GFP‐LC3 vesicles indicates enhanced autophagosome formation, inhibition of autophagosome clearance or both. Accordingly, to establish the high‐content fluorescence microscopy assay, the influence of the selected autophagy modulators on the GFP‐LC3‐positive vesicle area per cell was assessed using an automatic fluorescence microscope, followed by quantification with the analysis software (Fig. 1A). The results for the control treatments (fed, fed with rapamycin, starved and starved with chloroquine) of 16 independent experiments were summarised. Treatment with 1 μm rapamycin for 3 h resulted in a 1.9‐fold increase in the level of GFP‐LC3‐positive vesicles compared with the fed cells, nearly the same as the twofold increase observed after 1.5 h of serum and amino acid starvation (Fig. 1B). The addition of 60 μm chloroquine to starved cells elevated the level of GFP‐LC3‐positive vesicles approximately 4.7‐fold compared with the fed non‐treated cells and 2.4‐fold compared with starved, non‐treated cells due to the inhibition of autophagosome clearance (Fig. 1B). These results established our assay as sensitive and specific for detecting autophagy‐modulating compounds. The assay exhibited a *Z*′ factor of 0.75 and was therefore well suited for high‐throughput screening.

$$Z^{\prime }=1-\frac{\left(3{\mathrm{SD}}_{+}+3{\mathrm{SD}}_{-}\right)}{\left|{\mathrm{Ave}}_{+}-{\mathrm{Ave}}_{-}\right|}$$

$$Z^{\prime }=1-\frac{\left(3\cdot \left(\frac{1.83+2.56+1.79}{3}\right)+3\cdot \left(\frac{0.2+0.45+0.52}{3}\right)\right)}{\left|\left(\frac{41.34+38.28+35.13}{3}\right)-\left(\frac{9.39+9.52+8.75}{3}\right)\right|}$$

$$Z^{\prime }=1-\frac{\left(3\cdot 2.06+3\cdot 0.39\right)}{\left|38.25-9.22\right|}=0.75$$

Equation 1 shows the calculation of the *Z*′‐factor with SD_+_ being the standard deviation of all positive controls, SD_−_ the standard deviation of all negative controls, Ave_+_ the average of all positive controls, and Ave_−_ the average of all negative controls.

### Identification of chemical and natural compounds modulating autophagy

After establishing our read‐out system, the compound screen was performed in duplicate with compounds added to cells either in complete medium (fed, not induced) or amino acid‐ and serum‐free medium (starved, induced) and the relative changes in vesicular GFP‐LC3 were assessed in comparison with the respective negative controls (either fed or starved cells). The compounds that increased or decreased the amount of vesicular GFP‐LC3 by more than three standard deviations around the negative control were classified as hits.

First, we employed a small test library containing 35 chemical substances, including commercial drugs with known antimicrobial activities, to search for new properties and potential novel medical uses (re‐purposing). Under both fed (basal autophagy) and starved (induced autophagy) conditions, several compounds modulated autophagy in this initial screen (Fig. 1C and Table 1). For instance, the actin polymerisation inhibitor cytochalasin D increased the GFP‐LC3 vesicles under both conditions, consistent with previous reports [27]. CCCP, a mitochondrial uncoupler known to induce mitophagy [28], induced autophagy in fed cells but was cytotoxic to starved cells, as expected. The antifungal compound amphotericin B increased the amount of GFP‐LC3 vesicles in fed cells. Of note, the antifungicidal activity of amphotericin B was only reported in autophagy‐competent yeast cells, and amphotericin B was suggested to activate an alternative autophagy pathway in Atg5‐deficient cells [29, 30]. In contrast, luciferase inhibitor I and penicillin G were initially detected as hits, but their effects on autophagy could not be confirmed in follow‐up experiments (data not shown). Anisomycin and aciclovir decreased GFP‐LC3 vesicles (Table 1). The effect of the antiviral compound aciclovir was unexpected and prompted further investigation.

**Table 1:** Summary of primary hits found using the initial high‐throughput fluorescence screen of commercially available bioactive as well as myxobacterial and fungal compounds. In the screen, all compounds were used at a concentration of 10 μm. Compounds that were further characterised are indicated in bold. Disozarol and tubulysin class compounds exerted both increasing and decreasing effects on the GFP‐LC3 vesicles. Arrows indicate a constant (**→**), an increase in (**↑**) or a decrease (**↓**) in the total area of GFP‐LC3‐positive vesicles per cell.

Compound class	Compounds	Area GFP‐LC3 fed	Area GFP‐LC3 starved
**Aciclovir**		**↓**	**→**
Amphotericin B		**↑**	**↓**
**Anisomycin**		**↓**	**↓**
Archazolid	A, B	**↑**	**↑**
Apicularen	A, B	**↑**	**↑**
CCCP		**↑**	Toxic
Cohaerin	F	**↑**	**→**
Crocacin	D	**↑**	**↑**
Cytochalasin	D	**↑**	**↑**
Disorazol	A, A2, A3, A4, A5, A7, B, B2, B3, B4, D1, E1, Z	**↑**	**↑** **↓**
Epothilone	A, B, C, D, E		
Hyaboron	HYM3_BORKOMPLEX	**↑**	**↑**
Isochaetochromin	B1	**→**	**↑**
Luciferase Inhibitor I		**↓**	**↓**
Penicillin	G	**→**	**↑**
Noricumazol	A, B, C	**↑**	**↑**
Resveratrol		**↓**	**↓**
Rickiol	F	**↓**	**↓**
Tartolon	A, B, C	**↑**	**↓**
Trichothecolone acetate		**↓**	**↓**
Truncaton	C	**↑**	**↑**
Tubulysin	A N‐oxid, Ar672, B, C, G, W, X, Y, Z	**↑**	**↑** **↓**
**Vioprolide**	**A, B, C, D**	**↓**	**↓**

Additionally, we screened an in‐house library of approximately 300 natural compounds from myxobacteria and fungi, some of which have unique chemical scaffolds, to identify additional autophagy modulators (Fig. 1D,E, Table 1). While disorazol and tubulysin compounds, which bind to tubulin, were also identified as autophagy modulators, we excluded them from further study due to their known role in disrupting microtubule function, which is crucial for autophagosome trafficking [31]. Cohaerin F is an azaphilone compound isolated from the fungus *Annulohypoxylon cohaerens* [32]. In the initial screening experiment, cohaerin F increased the GFP‐LC3‐positive vesicle area in fed cells. However, since cohaerin F has to be purified from fungal fruiting bodies, the amounts available were too limited to allow for further testing in the course of this study. Additional primary hits are summarised in Table 1. Interestingly, we identified vioprolide A, B, C and D, secondary metabolites from myxobacteria that target Nucleolar Protein 14 (NOP14), a key factor in ribosome biogenesis [33], as compounds that decreased the area of GFP‐LC3‐positive vesicles in fed and starved cells. As vioprolide C and D had a prominent effect on autophagy, we selected these two compounds for further experiments.

Based on the results described above, we selected aciclovir, vioprolide C and vioprolide D for further testing because they have been well‐characterised but not connected to autophagy. Anisomycin was previously described as an autophagy inhibitor due to its effect on p38 MAPK signalling [34]; it was included as a positive control. None of the selected compounds negatively affected cell viability in fed nor in starved cells (Fig. 1F) at 10 μm, the concentration used for the initial screen.

### Validation of autophagy‐modulating compounds

**Fig. 2:**
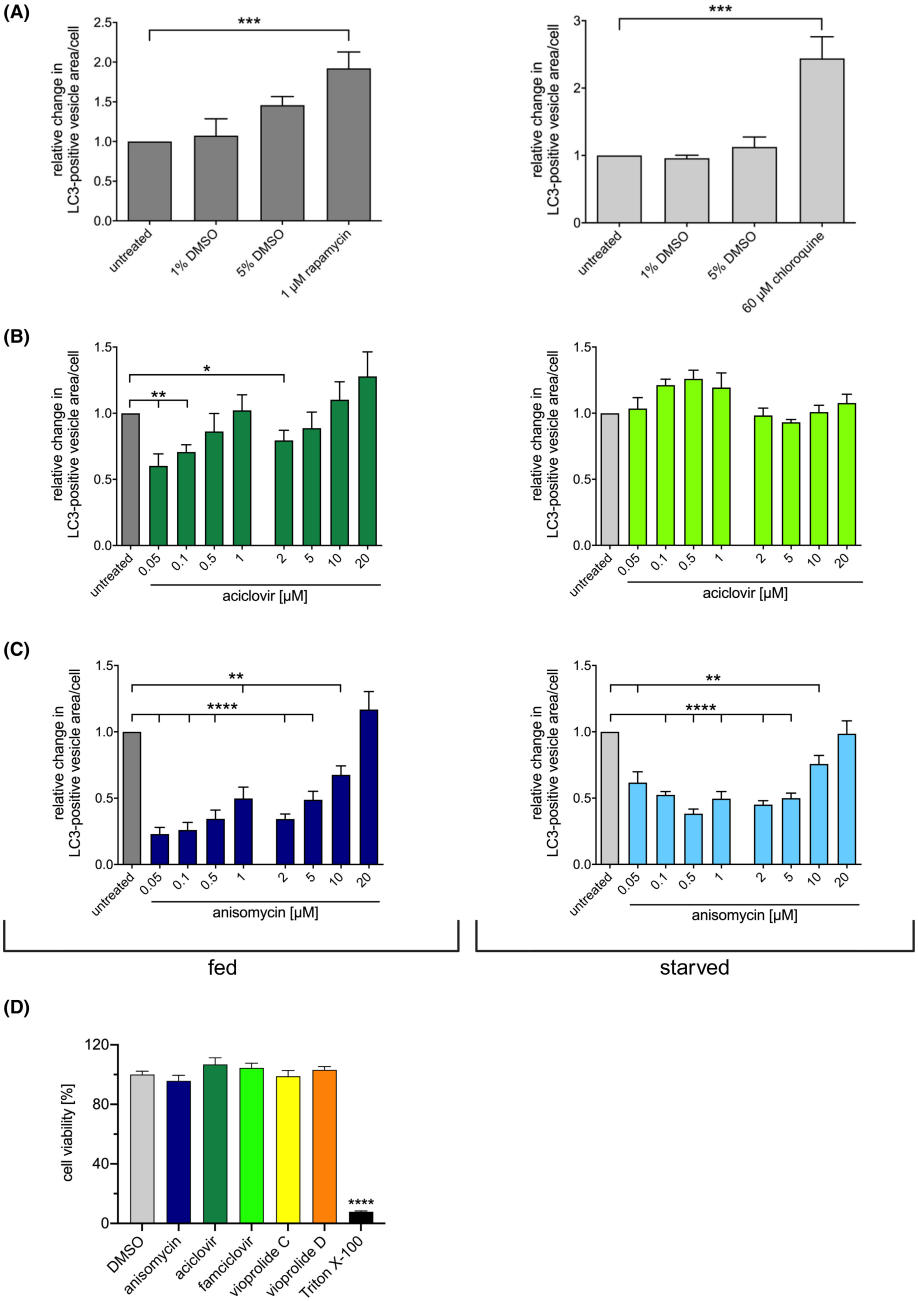
Dose–response effects of selected compounds from the commercial compound library on the level of GFP‐LC3‐positive vesicles. (A) Relative changes in the GFP‐LC3‐positive vesicle area per A549 cell caused by the selected control treatments compared with cells incubated in full medium without additions (untreated). Dimethyl sulfoxide (DMSO) was used to solubilise part of the compounds and used as a solvent control. (B) Concentration‐dependent effects of aciclovir on the GFP‐LC3‐positive vesicle area per cell in fed or starved A549 cells. Relative changes were calculated in comparison with the respective control (fed cells without compound (i.e., untreated) and starved cells without compound (i.e., untreated), respectively). Fed cells were cultivated for 3 h in full medium and starved cells for 1.5 h in serum and amino acid‐free medium. (C) Concentration‐dependent effects of anisomycin on the GFP‐LC3‐positive vesicle area per cell in fed or starved A549 cells. Analysis as in (B). Results in (B) and (C) are from two independent experiments. (D) A549 cells treated with 1 μm anisomycin, 1 μm aciclovir, 1 μm famciclovir, 5 μm vioprolide C and 5 μm vioprolide D for 3 h, and cell viability was measured by resazurin assay. DMSO served as a solvent control. Triton X‐100 was used as a positive control for cell death. Results are from three independent experiments done in triplicate. Bar graphs represent the mean; error bars represent SEM. Statistical indication according to unpaired two‐sided *t*‐test as follows: ****P < 0.0001, ***0.0001 < *P* < 0.001, **0.001 < *P* < 0.01, *0.01 < *P* < 0.05. Created in BioRender. Schmitz, I. (2025) https://BioRender.com/qk88jpv.

Next, we tested the autophagy‐modulating effects of aciclovir and anisomycin in more detail by analysing their dose–response characteristics. The solvent control, DMSO, did not affect autophagy, while the positive control substances rapamycin and chloroquine increased GFP‐LC3‐positive vesicles under fed and starved conditions, respectively (Fig. 2A).

Aciclovir decreased the amount of GFP‐LC3 vesicles in fed cells with decreasing compound concentrations (Fig. 2B), indicating that the compound either inhibits autophagosome formation or enhances autophagosome clearance at low concentrations. Hence, it was further titrated down to investigate the effects of even lower concentrations (0.05, 0.1, 0.5, 1 μm) in an additional experiment (Fig. 2B). In fed cells, a consistent decrease in the GFP‐LC3 vesicle area per cell was observed with lower concentrations of aciclovir. Interestingly, aciclovir did not change the level of GFP‐LC3 vesicles in starved cells, even though it decreased the GFP‐LC3 vesicles in fed cells at low concentrations (Fig. 2B), suggesting it only affects basal autophagy.

Anisomycin strongly decreased the amount of GFP‐LC3 vesicles in fed and starved cells and exhibited a dose dependency with stronger inhibition at lower concentrations (Fig. 2C). Induced autophagy was efficiently inhibited in starved cells at concentrations of 5 μm and lower (Fig. 2C). In fed cells, anisomycin had the strongest effect on the level of GFP‐LC3 vesicles at the lowest concentration tested, 0.05 μm (Fig. 2C).

To confirm that the observed changes in autophagy were not due to compound toxicity, we tested their effect on cell viability via resazurin assay. At 1 μm, aciclovir, anisomycin and the two vioprolides did not affect cell viability, similar to the DMSO control (Fig. 2D). In addition to aciclovir, we tested famciclovir as an additional guanosine derivative, which also did not affect cell viability. The positive control Triton X‐100 efficiently killed the cells (Fig. 2D).

### Effects of the hit compounds on autophagy flux and the co‐localisation of GFP‐LC3 with the lysosomal and late endosomal LAMP2

To further validate the effect of anisomycin, aciclovir, famciclovir and vioprolides C and D on autophagy, we used confocal microscopy to quantify the GFP‐LC3 vesicles and autophagy flux in the absence and presence of bafilomycin A1. Bafilomycin A1 prevents lysosome acidification by inhibiting the lysosomal H^+^‐ATPase; it also inhibits autophagosome‐lysosome fusion [35, 36]. Therefore, autophagosomes formed in bafilomycin A1‐treated cells are not cleared by lysosomal degradation.

**Fig. 3:**
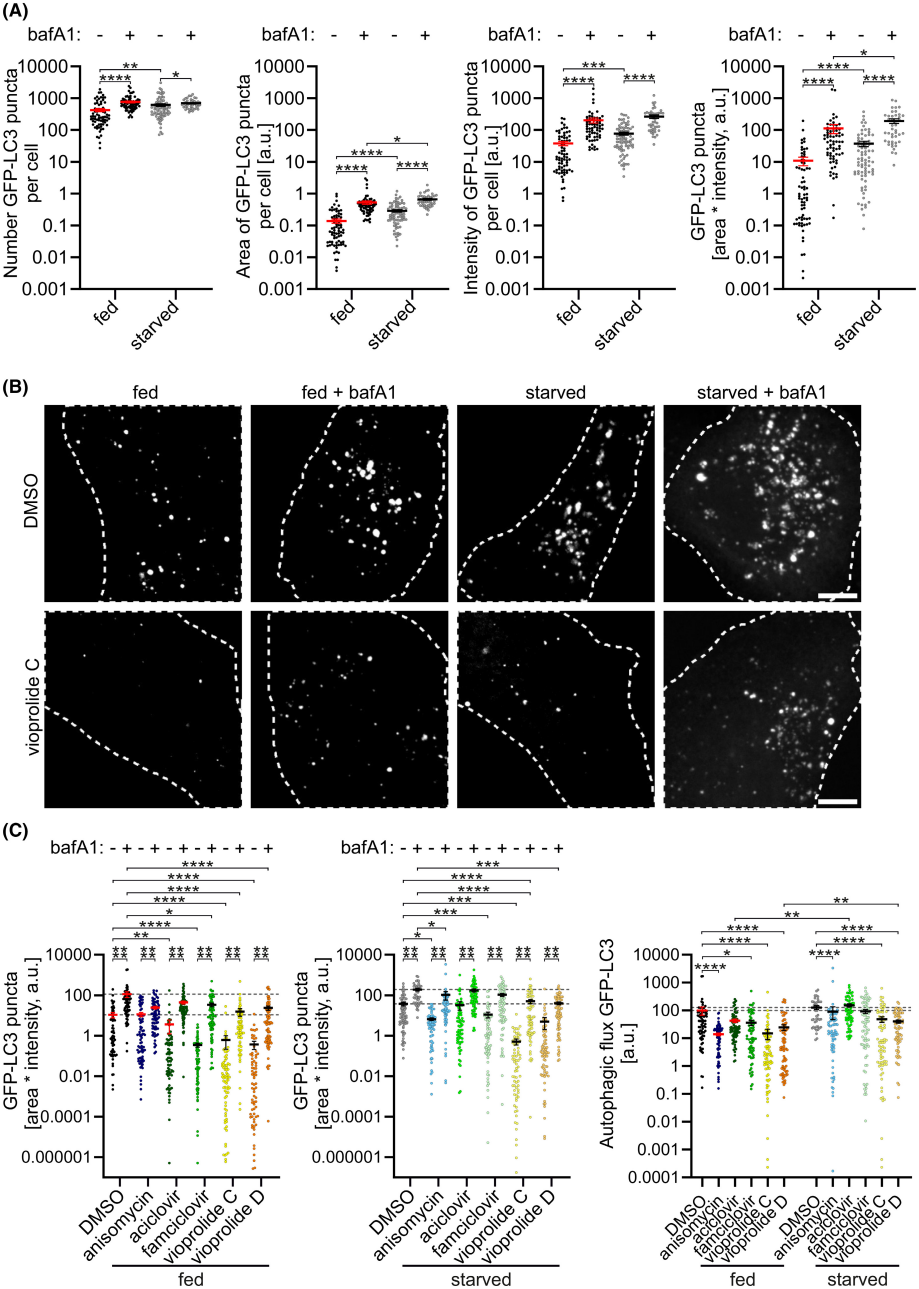
Effect of test compounds on GFP‐LC3 vesicles and autophagy flux in A549 cells. (A) Validation of GFP‐LC3 vesicle quantification by confocal microscopy. GFP‐LC3‐expressing A549 cells were incubated for 2 h in the absence or presence of 100 nm bafilomycin A1 (bafA1), either in full culture medium (fed), or serum and amino acid‐free medium (starved). GFP‐LC3‐positive vesicles were quantified using confocal microscopy as detailed in the Materials and methods section. (B, C) Quantification of the effects of the test compounds on GFP‐LC3 vesicles and autophagy flux. Cells were treated with 1 μm anisomycin, 1 μm aciclovir, 1 μm famciclovir, 5 μm vioprolide C and 5 μm vioprolide D for 2 h. (B) Representative images of maximum intensity projections of confocal microscopy images of cells under the four conditions, as indicated. Top row: Dimethyl sulfoxide (DMSO)‐treated control cells; bottom row: vioprolide C‐treated cells. Dashed lines indicate the approximate boundaries of individual cells. Scale bars: 5 μm. (C) Quantification of GFP‐LC3 vesicles, expressed as vesicle area multiplied by intensity, as well as GFP‐LC3 flux. The horizontal dotted lines indicate the mean of the DMSO‐treated control cells without and with bafilomycin A1. For the panels on the left and in the middle, horizontal dotted lines indicate the mean of the DMSO‐treated control cells without (lower line) and with bafilomycin A1 (upper line). For the panel on the right, dotted lines indicate the mean of fed (lower line) or starved (upper line) DMSO‐treated controls. A total of 41–78 (A) or 71–84 (C) cells from two independent experiments were analysed. The mean and standard error of the mean (SEM) for each sample are indicated. Kruskal–Wallis test and *post hoc* Dunn's multiple comparisons test were used to determine statistical significance: *P < 0.05, **P < 0.01, ***P < 0.001, ****P < 0.0001.

We validated the confocal microscopy quantification method for A549 cells under four conditions: full culture medium (fed) without and with bafilomycin A1 (basal autophagy), and serum and amino acid starvation (starved) without and with bafilomycin A1 for 2 h (amino acid starvation‐induced autophagy). The GFP‐LC3 vesicles were quantified in all optical sections for each cell. To determine the most accurate metric for GFP‐LC3 vesicle abundance per cell, we compared the count of GFP‐LC3 vesicles, total area of GFP‐LC3 vesicles, total intensity of GFP‐LC3 vesicles and total area multiplied by total intensity (Fig. 3A), all normalised to cell area. We found that total area multiplied by total intensity provided the most accurate results because it took into account variations in the size of the GFP‐LC3 vesicles: small and large vesicles contributed according to their area and intensity, not their number. We observed an increase in GFP‐LC3 vesicles under amino acid starvation (Fig. 3A) and, as expected, bafilomycin A1 treatment increased the level of GFP‐LC3 vesicles under both fed and starved conditions.

The effects of the test compounds were then analysed using confocal microscopy. GFP‐LC3 vesicles were quantified under fed and starved conditions, both in the absence and presence of bafilomycin A1 (Fig. 3B,C). Bafilomycin A1 significantly increased the amount of GFP‐LC3 vesicles for all treatments, as expected. Compared with DMSO treatment, anisomycin decreased GFP‐LC3 vesicles only under starvation, while aciclovir inhibited GFP‐LC3 vesicle accumulation only under fed conditions in the absence of bafilomycin A1. Famciclovir decreased the vesicles under fed conditions and under starved conditions when bafilomycin A1 was not present. Vioprolide C decreased the vesicles under all conditions, while vioprolide D inhibited the vesicle accumulation under fed conditions and under starved conditions when bafilomycin was present.

Autophagy flux is an indicator of the function of the entire autophagy pathway, from autophagosome formation to the degradation of the cytoplasmic cargo [7]. GFP‐LC3 flux was calculated by subtracting the GFP‐LC3 puncta values of samples without bafilomycin A1 from those of the corresponding samples with bafilomycin A1 (Fig. 3C, right). Anisomycin and both vioprolides reduced GFP‐LC3 flux under both fed and starved conditions, while famciclovir only reduced the flux under fed conditions. Because anisomycin did not significantly reduce the amount of GFP‐LC3 vesicles in fed cells, the reduced flux in anisomycin‐treated fed cells is likely due to reduced clearance of the vesicles. Aciclovir did not change GFP‐LC3 flux compared with the DMSO‐treated controls. However, under starvation, both aciclovir and vioprolide D increased autophagic flux under starvation conditions compared with fed conditions.

**Fig. 4:**
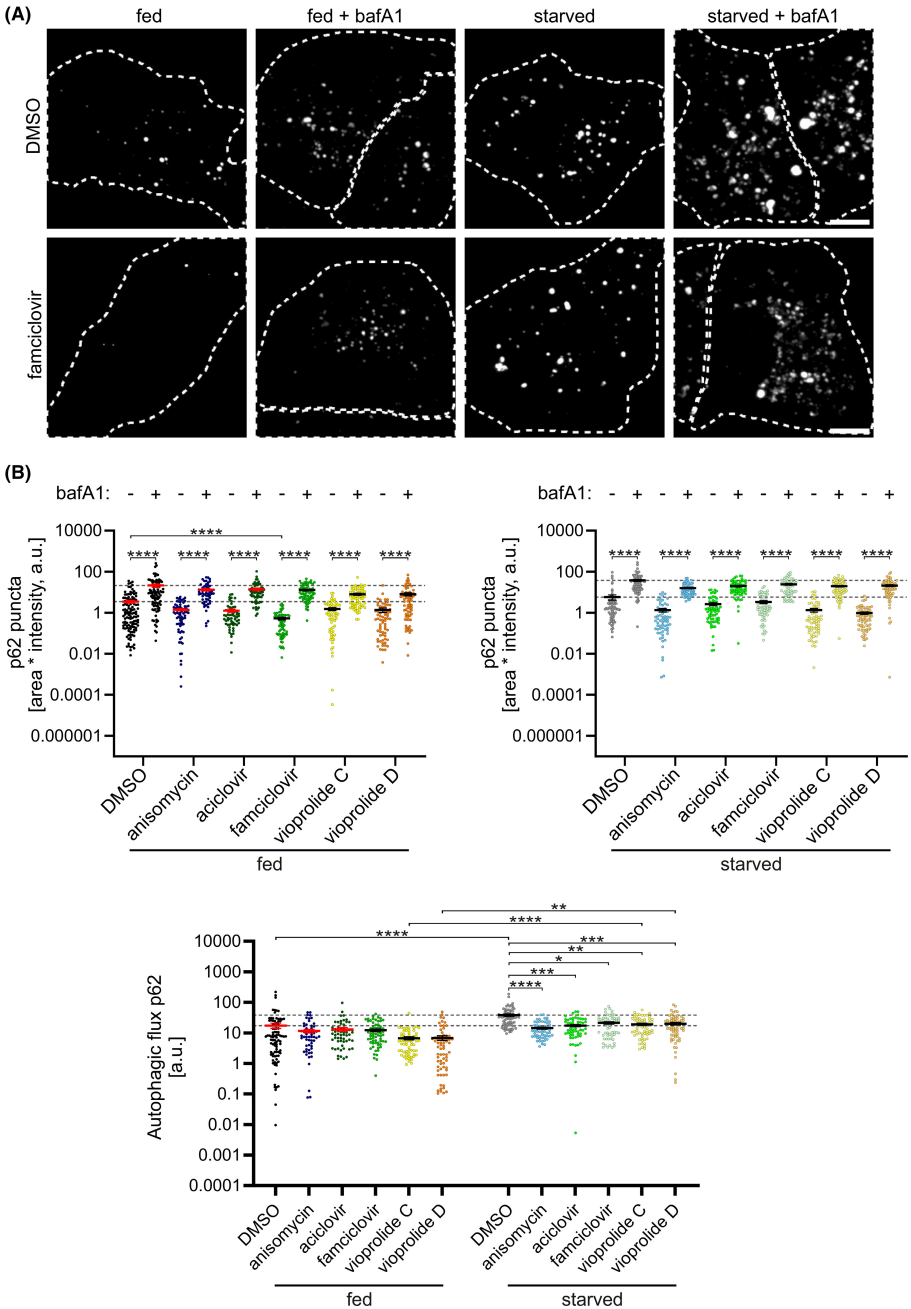
Quantification of endogenous SQSTM1/p62 puncta and autophagy flux in A549 cells. A549 cells were treated for 2 h with 1 μm anisomycin, 1 μm aciclovir, 1 μm famciclovir, 5 μm vioprolide C and 5 μm vioprolide D, in full medium (fed) or serum and amino acid‐free medium (starved), without or with 100 nm bafilomycin A1 (bafA1). Cells were fixed and stained for endogenous p62, and puncta were quantified by confocal microscopy. (A) Representative images showing maximum intensity projections of cells treated with DMSO or famciclovir. Dashed lines indicate the approximate boundaries of individual cells. Scale bars: 5 μm. (B) Quantification of p62 puncta and flux by confocal microscopy. The horizontal dotted lines indicate the mean of the dimethyl sulfoxide (DMSO)‐treated cells without and with bafilomycin A1. For the panels on the left and in the middle, the horizontal dotted lines indicate the mean of the DMSO‐treated cells without (lower line) and with bafilomycin A1 (upper line). For the panel on the right, dotted lines indicate the mean of fed (lower line) or starved (upper line) DMSO‐treated controls. For each sample, a total of 71–84 cells from two independent experiments were analysed. The mean and standard error of the mean (SEM) for each sample are indicated. Kruskal–Wallis test and *post hoc* Dunn's multiple comparisons test were used for statistical significance. *P < 0.05, **P < 0.01, ***P < 0.001, ****P < 0.0001.

Sequestosome 1 (SQSTM1), also called p62, is one of the autophagy receptor proteins that is degraded together with the cargo in selective autophagy [37]. In addition, p62 forms condensates that initiate autophagosome formation in non‐selective autophagy [38, 39]. Thus, similar to LC3 puncta, p62 puncta can be used to monitor autophagy. We used the confocal microscopy approach to assess the effects of our test compounds by immunofluorescence staining of endogenous p62 in A549 cells. As expected, bafilomycin A1 increased p62 puncta in all treatments (Fig. 4A,B). Compared with the DMSO control, all tested compounds showed a tendency to decrease p62 puncta both in the absence and presence of bafilomycin A1, but the differences only reached statistical significance for famciclovir in fed cells when bafilomycin A1 was absent. Furthermore, all test compounds decreased p62 flux both under fed and starved conditions, but compared with the DMSO‐treated cells, the differences were statistically significant only under starved conditions (Fig. 4B, right). Moreover, in cells treated with DMSO, vioprolide C or vioprolide D, p62 flux was significantly higher under starvation than under fed conditions (Fig. 4B, lower).

**Fig. 5:**
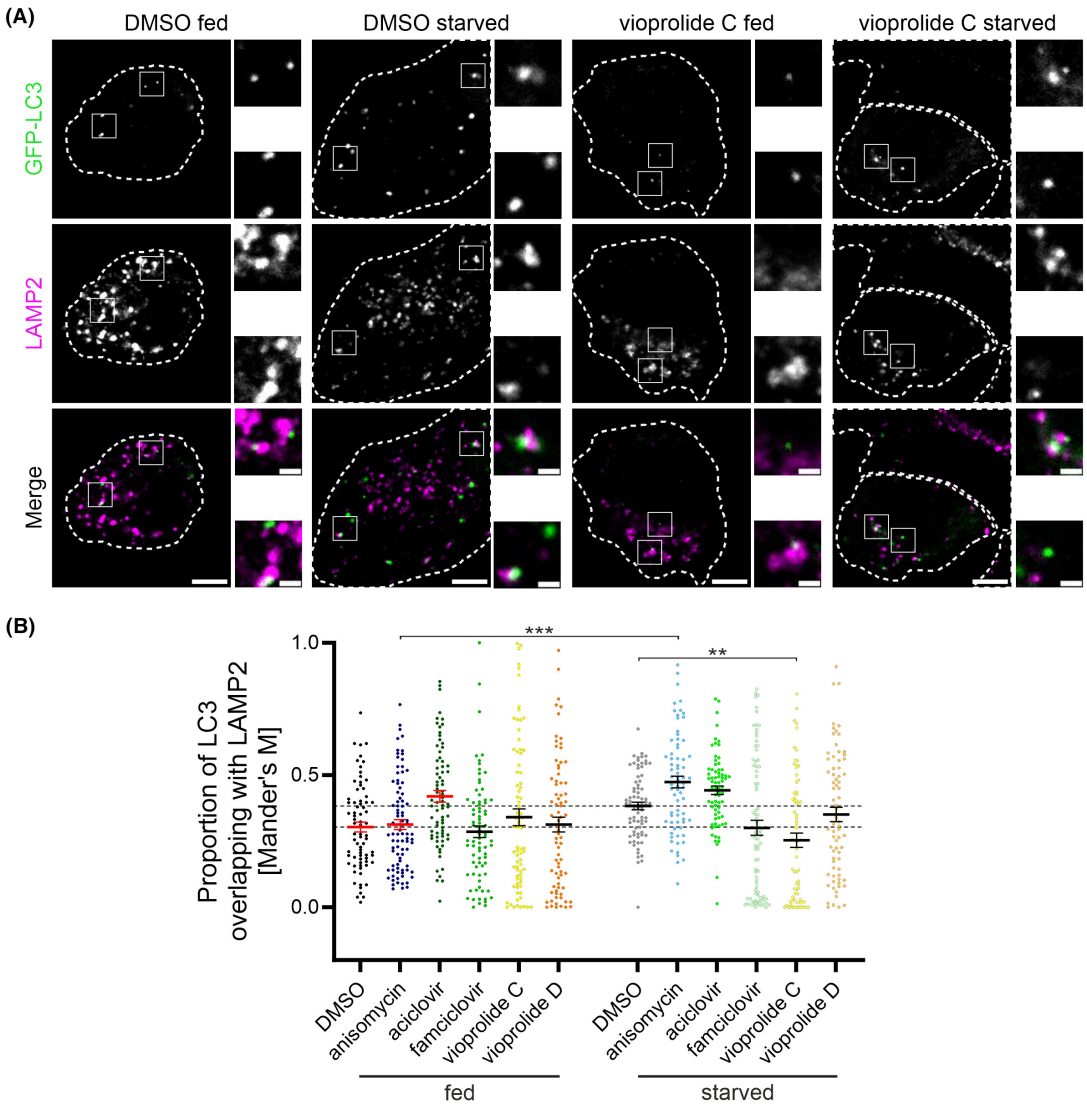
Effect of the test compounds on autophagosome fusion with LAMP2‐positive compartments. GFP‐LC3‐expressing A549 cells were treated with the test compounds for 2 h (1 μm anisomycin, 1 μm aciclovir, 1 μm famciclovir, 5 μm vioprolide C and 5 μm vioprolide D) in full medium (fed) or in serum and amino acid‐free medium (starved). Cells were fixed and stained for endogenous LAMP2. Co‐localisation of GFP‐LC3 with LAMP2 was analysed using confocal microscopy, including all optical sections for each cell in the analysis. (A) Representative images of individual optical sections from fed and starved control [dimethyl sulfoxide (DMSO)] and vioprolide C‐treated cells. Dashed lines indicate the approximate boundaries of individual cells. Scale bars: 5 μm for the overview image and 1 μm for the magnified section. (B) Proportion of GFP‐LC3‐positive signals that overlap with LAMP2 signals, expressed as Mander's coefficient. The horizontal dotted lines indicate the mean of the DMSO‐treated cells without and with bafilomycin A1; the horizontal dotted lines indicate the mean of fed (lower line) and starved (upper line) DMSO‐treated cells. A total of 71–84 cells per condition from two independent experiments were analysed. The mean and standard error of the mean for each sample are indicated. Kruskal–Wallis test and *post hoc* Dunn's multiple comparisons test were used for statistical significance: **P < 0.01, ***P < 0.001.

Finally, we used confocal microscopy to analyse autophagosome fusion with LAMP2‐positive compartments. LAMP2 is a lysosomal membrane protein that is also present in late endosomes. GFP‐LC3‐expressing A549 cells were stained for endogenous LAMP2, and the percentage of GFP‐LC3 signals positive for LAMP2 were analysed using Mander's coefficient (Fig. 5A,B). Compared with DMSO, none of the tested compounds significantly affected GFP‐LC3‐LAMP2 co‐localisation under fed conditions (Fig. 5B). This suggests that, under fed conditions, they do not inhibit autophagosome fusion with LAMP2‐positive compartments. Under starved conditions, vioprolide C significantly decreased GFP‐LC3‐LAMP2 co‐localisation (Fig. 5A,B), suggesting that at least part of its autophagy inhibition was due to inhibition of autophagosome fusion with LAMP2‐positive compartments. In anisomycin‐treated cells, the proportion of GFP‐LC3 signals overlapping with those of LAMP2 signals was significantly higher under starved conditions compared with fed conditions (Fig. 5B).

To summarise the results of the confocal microscopy analysis, anisomycin decreased GFP‐LC3 vesicles in starved cells, decreased GFP‐LC3 flux in both fed and starved cells, and p62 flux in starved cells. In anisomycin‐treated cells, starvation increased the co‐localisation of GFP‐LC3 and LAMP2. These results suggest that anisomycin likely inhibits both autophagosome formation and clearance. Famciclovir decreased GFP‐LC3 vesicles in fed and starved cells, GFP‐LC3 flux in fed cells and p62 flux in starved cells, but it did not significantly alter co‐localisation of GFP‐LC3 with LAMP2. These results suggest that famciclovir inhibits autophagosome formation. Vioprolide C and vioprolide D decreased GFP‐LC3 vesicles in fed and starved cells, GFP‐LC3 flux in both fed and starved cells, and p62 flux in starved cells. Vioprolide C also decreased GFP‐LC3‐LAMP2 co‐localisation in starved cells, while vioprolide D did not significantly change the co‐localisation. This suggests that both vioprolides inhibit autophagosome formation, while vioprolide C may also inhibit autophagosome fusion with LAMP2‐positive compartments under starved conditions.

### Effects of autophagy‐modulating compounds on bacterial and viral infection

**Fig. 6:**
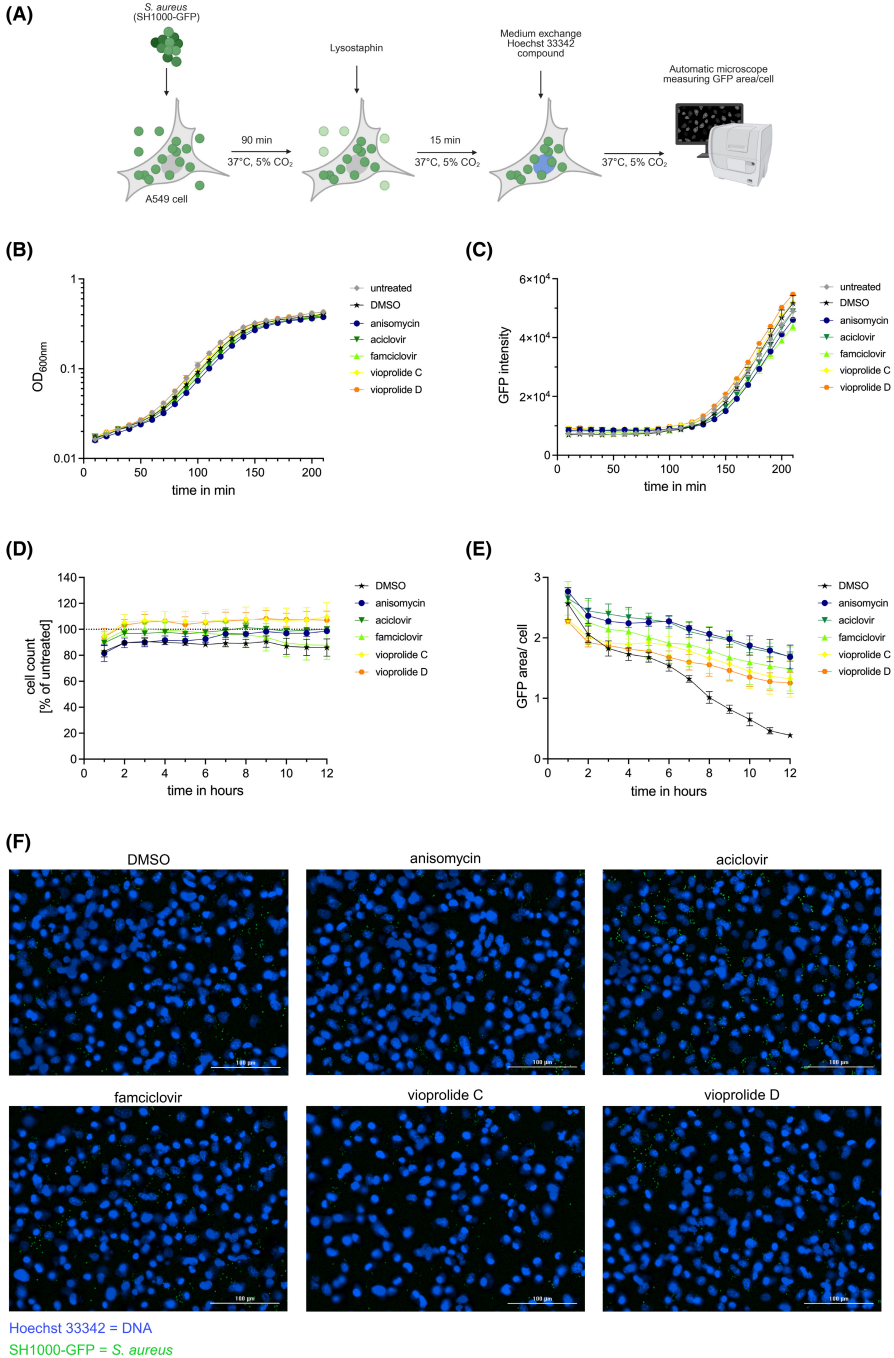
Autophagy inhibitors promote survival of intracellular *S. aureus*. (A) Schematic representation of the experimental design. A549 cells were incubated with *S. aureus* SH1000‐GFP for 90 min at +37 °C. Then, all extracellular bacteria were killed by adding lysostaphin. After a 15‐min incubation at +37 °C, the medium was changed. The nuclear DNA was stained with Hoechst 33342, and the cells were treated either with 10 μm of the indicated compounds or dimethyl sulfoxide (DMSO) as solvent control. A picture was taken every hour, starting at time point 0 h postinfection (hpi), and GFP area/cell was measured using the pictures. (B, C) Bacterial growth curves. *S. aureus* SH1000‐GFP cultures were treated with either 10 μm of the indicated compounds or DMSO. The bacterial growth was monitored by measuring OD_600_ (B) or GFP fluorescence intensity (C). (D) A549 cell viability as measured by cell counting via Hoechst 33342 staining of nuclear DNA. (E) Quantification of the experiment shown in (A). Shown is the GFP fluorescence area/cell over a time course of 12 h. Data are represented as mean ± SD of three independent experiments. Three wells were analysed per experiment. (F) Representative images of cells infected and treated as depicted in (A). Hoechst 33342‐stained DNA (blue); *S. aureus* strain SH1000‐GFP (green); Scale bars, 100 μm. Created in BioRender. Schmitz, I. (2025) https://BioRender.com/qk88jpv.

As it has been described that autophagy can act as an antimicrobial mechanism, also called xenophagy, we addressed whether our identified compounds modulated responses against intracellular pathogens. Inhibition of xenophagy would thus result in a higher pathogen burden. We have previously reported that xenophagy is induced during *S. aureus* infection and that the bacteria counteract this process by exploiting the host p38 MAP kinase [18]. Notably, anisomycin is known to induce the activation of p38 MAP kinase. Therefore, we infected A549 cells with GFP‐expressing *S. aureus* for 90 min, killed all extracellular bacteria with lysostaphin and incubated the infected cells in the presence of anisomycin, aciclovir, famciclovir or the vioprolides for up to 12 h (Fig. 6A). Equal concentrations of these compounds did not affect *S. aureus* growth when added to liquid bacterial cultures, as measured by optical density (OD_600_; Fig. 6B) and GFP fluorescence intensity (Fig. 6C). Moreover, the compounds did not significantly affect cell viability compared with the solvent control (Fig. 6D). In infection experiments, cells treated with aciclovir or anisomycin had a much higher bacterial load, as shown by a higher GFP fluorescence intensity compared with the solvent control (Fig. 6E,F). Similarly, the aciclovir analogue famciclovir and the vioprolides increased the bacterial load, albeit to a lesser extent (Fig. 6E,F). Our data suggest that inhibition of autophagy by aciclovir, anisomycin, famciclovir, vioprolide C or vioprolide D prevents bacterial elimination by xenophagy.

**Fig. 7:**
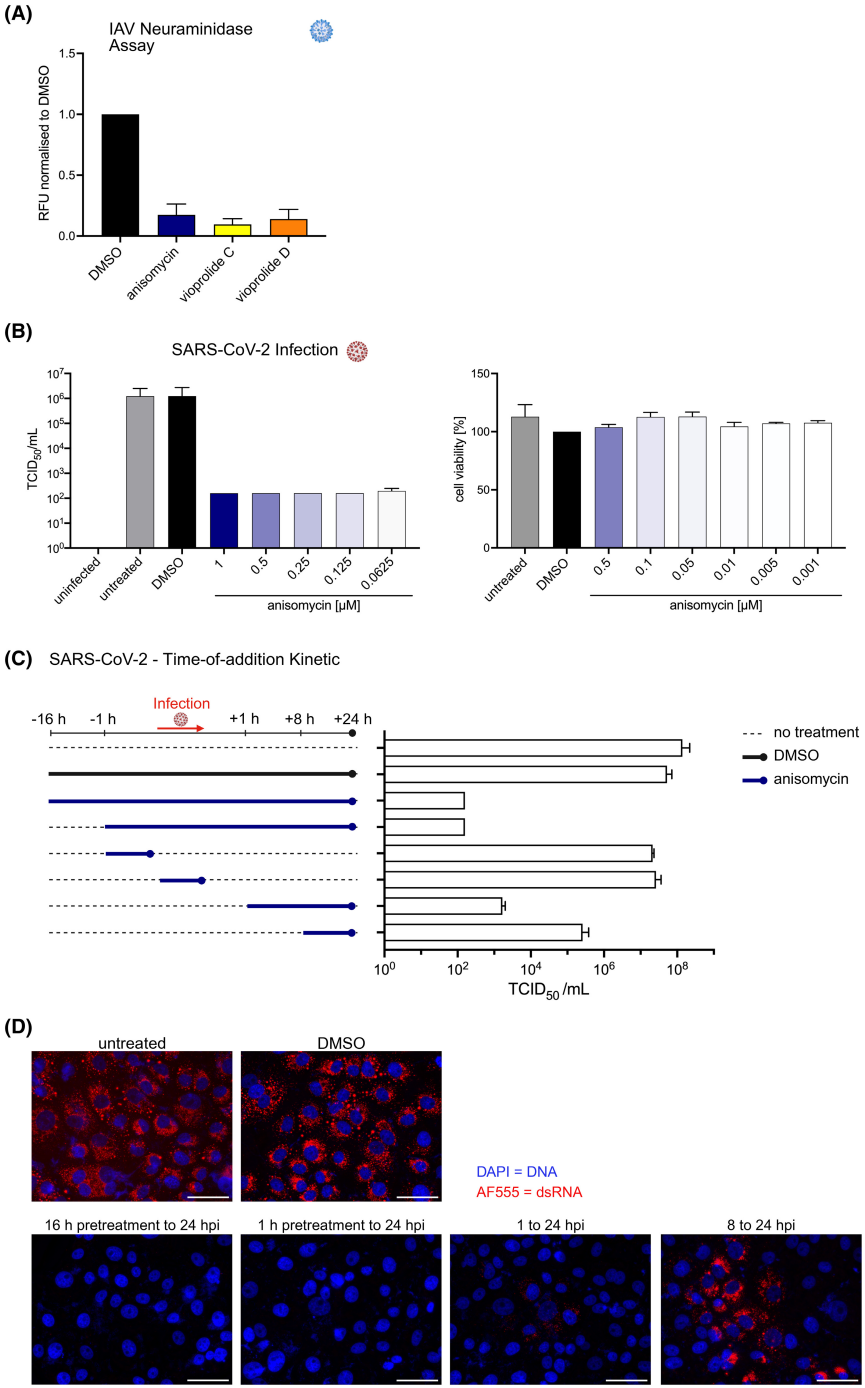
Effects of anisomycin and vioprolides C and D on Influenza A and SARS‐CoV‐2 replication. (A) A549 cells were infected with influenza A virus at a MOI of 2 for 1 h. After changing the medium, production media containing the test compounds at 10 μm were then added for 16 h at +37 °C. To monitor viral replication, a sensitive, broad‐range luminometric NA‐STAR assay was used for the determination of neuraminidase (NA) activity. Values are given in NA light units/well (RFU), measured from fourfold diluted supernatants. Results are from two experiments done in duplicate. Bar graphs represent the mean; error bars represent SEM. (B) VeroE6 cells were seeded in a 24‐well plate. After the cells had attached to the plate, they were treated with different concentrations of anisomycin for 1 h. The cells were then infected with SARS‐CoV‐2 (MOI 1). After 1 h, the cells were washed and treated again with anisomycin as indicated. One day postinfection, the supernatant was collected, and infectious viral titres were determined using an end‐point dilution assay on VeroE6 cells seeded in a 96‐well plate to calculate the 50% tissue culture infectious dose (TCID_50_)·mL^−1^. Cell viability was monitored by MTT assay. (C) Time‐of‐addition kinetics for anisomycin. VeroE6 cells were seeded in a 24‐well plate and treated with 0.1 μm anisomycin for different time periods, starting from 16 h preinfection to 8 h postinfection. The cells were infected with SARS‐CoV‐2 (MOI 1) for 1 h. The cells were then washed thrice and again treated with anisomycin as indicated. One day postinfection, the supernatant was collected, and infectious viral titres were determined by an end‐point dilution assay on VeroE6 cells. (D) Representative immunofluorescence images of the experiment shown in panel (C). DAPI‐stained DNA (blue); dsRNA (red); hpi, hours postinfection; scale bar, 50 μm. Results for (B–D) are from three experiments done in duplicate. Bar graphs represent the mean; error bars represent SD. Created in BioRender. Schmitz, I. (2025) https://BioRender.com/qk88jpv.

We then tested whether these compounds also influenced viral replication. Influenza A virus (IAV) has also been reported to modulate autophagy [40, 41, 42]. Thus, we tested anisomycin and vioprolide C and D, none of which were reported as being antiviral before, for their effects on the replication of IAV. To this end, we infected Madin‐Darby canine kidney (MDCK) epithelial cells with IAV in the presence of the inhibitors or solvent control and measured neuraminidase activity by chemiluminescence. Neuraminidase is an enzyme expressed on the surface of IAV virions and is required to release new IAV particles from an infected cell. It can thus serve as a measure for viral replication. At a concentration of 10 μm, anisomycin and both vioprolides strongly reduced neuraminidase activity, suggesting that the production of new viral particles was inhibited (Fig. 7A).

Given the emerging role of autophagy in SARS‐CoV‐2 infections [43], we investigated the effects of selected compounds on the replication of SARS‐CoV‐2. Aciclovir and famciclovir did not affect SARS‐CoV‐2 replication, while vioprolides had cytotoxic effects on VeroE6 cells (data not shown). In contrast, anisomycin strongly inhibited SARS‐CoV‐2 replication at all concentrations tested, as analysed by quantification of viral RNA copy numbers via qPCR (data not shown) and by 50% tissue culture infectious dose (TCID_50_) assay (Fig. 7B, left panel). Anisomycin did not affect cell viability at the concentrations tested (Fig. 7B, right panel). The strongest inhibition of SARS‐CoV‐2 replication was observed when the cells were pretreated for 1 or 16 h with 10 μm anisomycin, which was also present during and after infection (Fig. 7C). The presence of anisomycin for just 1 h before or during the infection process was not sufficient to inhibit virus infection, suggesting that anisomycin likely targets virus replication. Of note, adding anisomycin for 1 or 8 h postinfection resulted in reduced virus replication by three and five orders of magnitude, respectively (Fig. 7C). This was also visible by immunofluorescence microscopy when dsRNA, a replication intermediate of ssRNA viruses, was stained (Fig. 7D). Similar results were obtained in Calu‐2 cells (data not shown).

In summary, our infection experiments suggest modulation of autophagy as a potential therapeutic option for infection by *S. aureus* or respiratory viruses. Intracellular survival of *S. aureus* was enhanced by autophagy inhibition, indicating that activators of autophagy should be tested as potential anti‐infectives in the future. Anisomycin might be useful for the treatment of SARS‐CoV‐2 and potentially other coronaviruses.

### Effects of anisomycin, aciclovir, famciclovir, vioprolide C and vioprolide D on autophagy in Lagotto Romagnolo fibroblasts

In order to test the identified compounds in a non‐infectious disease context, we employed fibroblasts from Lagotto Romagnolo (LR) dogs. In addition to wild‐type (WT) cells, we used cells from dogs carrying a mutation in the ATG4D gene (c.1288G>A; p. A430T), which were reported to have elevated levels of LC3‐positive vesicles under basal conditions [14]. Moreover, this mutation is associated with neurodegenerative vacuolar storage disease (NVSD) [13]. The phenotype includes behavioural changes, mild atrophy of the cerebellum and forebrain, cerebellar ataxia, and loss of Purkinje and granular cells. Vacuoles were observed in neuronal cells and secretory epithelial cells. Vesicles positive for LC3B and autophagy cargo were observed in axonal spheroids and in a perinuclear area in Purkinje cells, suggesting altered autophagy in the affected neurons [13]. The cellular fibroblast model was employed to study putative disease‐modifying activities of our compounds.

For the analysis of autophagy activity in LR fibroblasts, we validated immunofluorescence staining of endogenous LC3 followed by quantification of the LC3‐positive vesicles as a proxy to autophagosomes [7]. As before, we validated this approach for LR dog fibroblasts using wild‐type (WT) cells under four conditions: full culture medium without and with bafilomycin A1 for 2 h (basal autophagy) and serum and amino acid starvation without and with bafilomycin A1 for 2 h (amino acid starvation‐induced autophagy). Using confocal microscopy, we compared the count of LC3 vesicles, total area of LC3 vesicles, total intensity of LC3 vesicles, and total area multiplied with total intensity, all normalised to cell area (data not shown). Similar to the results obtained in A549 cells, we found that total area multiplied with total intensity provided the most accurate results. We observed an increase in LC3 vesicles under amino acid starvation, and as expected, bafilomycin A1 treatment increased the level of LC3 vesicles under both basal and starvation conditions. This analysis confirmed that quantitative LC3 immunofluorescence can be used to monitor autophagy in LR fibroblasts.

**Fig. 8:**
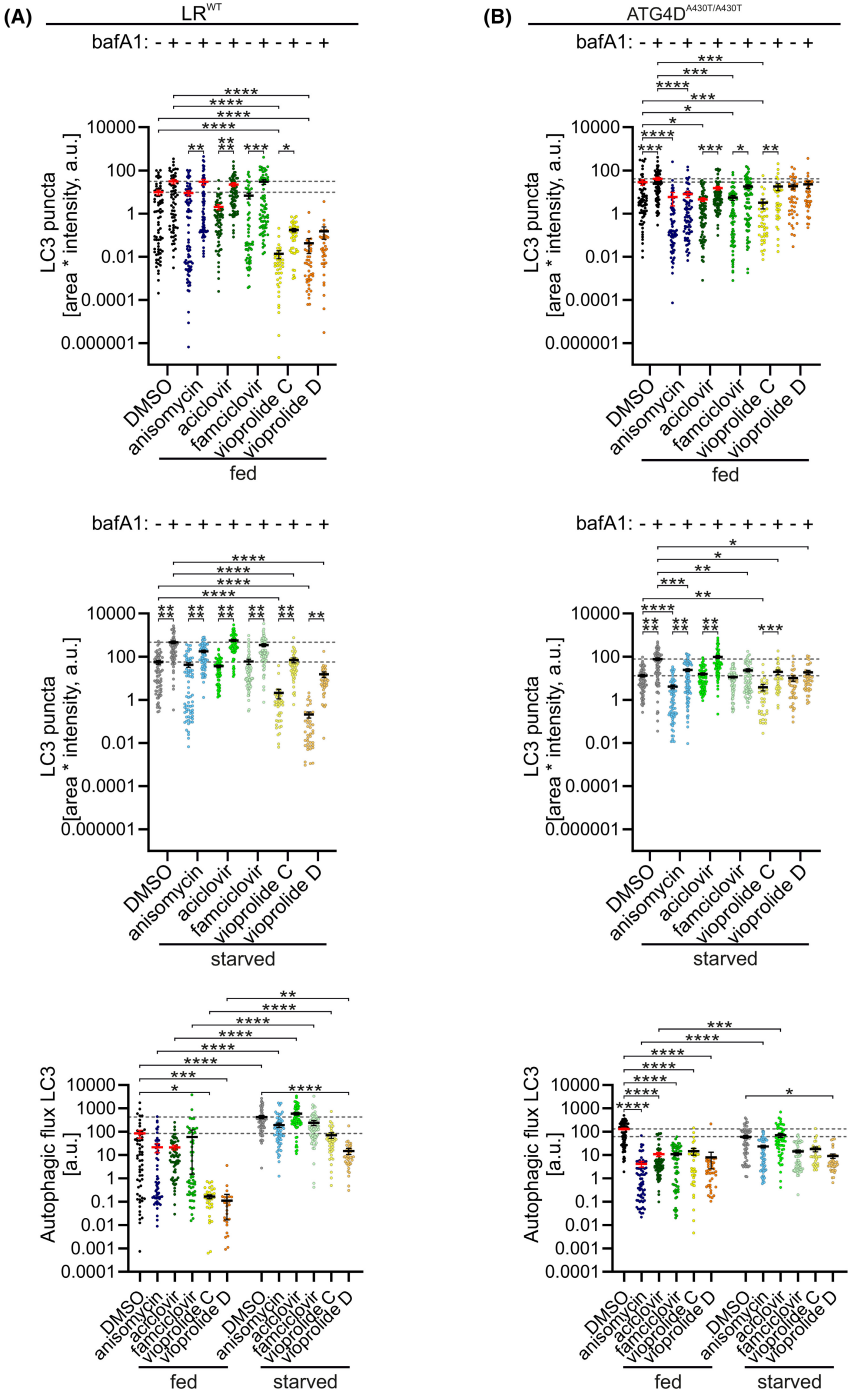
Quantifications of LC3‐positive vesicles detected by LC3 immunofluorescence staining in WT and ATG4D^A430T/A430T^ LR dog fibroblasts following treatment with candidate compounds. Cells were treated with dimethyl sulfoxide (DMSO), 1 μm anisomycin, 1 μm aciclovir, 1 μm famciclovir, 5 μm vioprolide C or 5 μm vioprolide D for 2 h, without or with 100 nm bafilomycin A1 (bafA1), under the indicated conditions, fed (full culture medium) or starved (serum and amino acid‐free medium). The cells were stained for endogenous LC3, and the amount of LC3‐positive vesicles and LC3 flux were quantified as described in Fig. 3. (A) WT LR fibroblasts. For the panels on the top and middle, the horizontal dotted lines indicate the mean of the DMSO‐treated controls without (lower line) and with bafilomycin A1 (upper lines). For the lower panel, dotted lines indicate the mean of fed (lower line) or starved (upper line) DMSO‐treated control WT cells. (B) ATG4D^A430T/A430T^ LR fibroblasts. The horizontal dotted lines indicate the mean values of the DMSO‐treated controls without and with bafilomycin A1. For the panels on the top and middle, the horizontal dotted lines indicate the mean of the DMSO‐treated controls without (lower line) and with bafilomycin A1 (upper lines). For the lower panel, dotted lines indicate the mean of fed (upper line) or starved (lower line) DMSO‐treated control ATG4D^A430T/A430T^ cells. For each condition in (A) and (B), a total of 40–84 cells from two independent experiments were analysed. The mean and standard error of the mean (SEM) for each sample are indicated. Kruskal–Wallis test and *post hoc* Dunn's multiple comparisons test were used for statistical significance: *P < 0.05, **P < 0.01, ***P < 0.001, ****P < 0.0001.

Next, we quantified LC3 vesicles in WT and ATG4D^A430T/A430T^ mutant LR fibroblasts, the latter of which were reported to have altered basal autophagy [14]. We observed that under fed conditions without bafilomycin A1, ATG4D^A430T/A430T^ cells indeed had higher levels of LC3 vesicles compared with the WT cells (Fig. 8A,B). This difference was not observed after 2‐h amino acid starvation, as the ATG4D^A430T/A430T^ cells had fewer LC3 vesicles than the WT cells, suggesting that during starvation, the ATG4D^A430T/A430^ cells did not upregulate autophagy as effectively as the WT cells. This was also seen in the comparison of autophagy flux: WT cells increased their LC3 flux during starvation, while ATG4D^A430T/A430^ cells did not.

Subsequently, anisomycin, aciclovir, famciclovir, vioprolide C and vioprolide D were investigated for their potential to modulate basal and starvation‐induced autophagy in WT and ATG4D^A430T/A430^ cells. Aciclovir was tested at two concentrations (1 and 0.5 μm). Because no differences were observed between these concentrations, the results are shown for the 1 μm concentration only. In fed WT cells, vioprolide C or vioprolide D strongly reduced the amount of LC3‐positive vesicles compared with DMSO (Fig. 8A). Similar inhibition was observed under serum and amino acid starvation in WT cells. These results suggest that vioprolide C and D strongly inhibit autophagosome formation in both fed and starved cells.

In fed ATG4D^A430T/A430T^ cells, anisomycin, aciclovir, famciclovir and vioprolide C slightly reduced LC3‐positive vesicles under fed conditions (Fig. 8B), suggesting slightly reduced autophagosome formation under basal conditions. Notably, under amino acid starvation, the reduction was observed only with anisomycin both with and without bafilomycin A1, and famciclovir, vioprolide C and vioprolide D with bafilomycin A1. Thus, anisomycin, famciclovir, vioprolide C and vioprolide D showed a slight inhibition of autophagosome accumulation also under amino acid starvation, while aciclovir did not affect starvation‐induced autophagy.

In WT cells, LC3 flux was significantly higher in starved cells than in the corresponding fed cells (Fig. 8A, bottom), indicating that none of the drugs prevented starvation‐induced increase in LC3 flux. Compared with DMSO, the flux was reduced by vioprolide C and vioprolide D under fed conditions, and by vioprolide D under starved conditions, in agreement with the large reduction in LC3 vesicles. These results suggest reduced autophagosome formation. Thus, vioprolide C and vioprolide D emerged as potent inhibitors of both basal and starvation‐induced autophagy flux in WT LR fibroblasts.

In fed ATG4D^A430T/A430T^ cells cultured in full medium, LC3 flux was decreased by all tested compounds (Fig. 8B, bottom); while in starved ATG4D^A430T/A430T^ cells, only vioprolide D reduced the flux. Notably, under amino acid starvation conditions, anisomycin did not reduce autophagic flux significantly, despite reduced levels of LC3‐positive vesicles both without and with bafilomycin A1 (Fig. 8B, middle).

The analysis also suggested elevated LC3 flux in ATG4D^A430T/A430T^ cells (130.9 ± 11.9) compared with WT LR cells (84.3 ± 31.2) under fed conditions; while under starvation, the flux was considerably lower in ATG4D^A430T/A430T^ cells (61.1 ± 7.0) compared with the WT LR cells (428.5 ± 67.5) (Fig. 8A,B, bottom). Under fed conditions, anisomycin, aciclovir, famciclovir and vioprolides C and D all decreased the elevated flux in ATG4D^A430T/A430T^ cells below that observed in DMSO‐treated WT LR cells.

## Discussion

Here, we describe the development of a high‐content microscopy‐based screening assay for autophagy modulation and its application to compound libraries. As a proof of principle, we first tested a small library of chemical compounds with known activities such as antibiotic or anti‐cancer properties as well as kinase or protein synthesis inhibition, which resulted in the identification of aciclovir and anisomycin as modulators of autophagy. Moreover, we screened a small in‐house library of natural compounds of myxobacterial and fungal origin. Regarding these natural compounds, we focused on vioprolide C and vioprolide D, which had been shown to have anti‐cancer and antifungal activities [44]. Based on our findings, we wondered whether new applications could be found for these ‘old drugs’, as the procedure known as repurposing or repositioning is often used in pharmaceutical research [20]. Thus, we further investigated the influence of the autophagy modulators on the replication of *S. aureus*, Influenza A, and SARS‐CoV‐2 as well as in a cellular disease model of NVSD.

### Aciclovir and famciclovir

Aciclovir and its guanosine analogues are approved anti‐herpesvirus drugs, used to treat, for example, Herpes Simplex Virus‐1, Herpes Simplex Virus‐2, Varicella zoster, Epstein–Barr Virus or cytomegalovirus infections. The mode of their action and side effects are well investigated. Aciclovir specifically interferes with viral DNA replication [45]. We found that aciclovir and/or its derivative famciclovir decreased basal autophagy in A549 cells but had a less pronounced effect on autophagy in starved cells, indicating a novel mechanism of interference with the autophagy pathway. For this reason, it was interesting to investigate the effects in the NVSD model, in which basal autophagy but not induced autophagy is affected [14]. Aciclovir and famciclovir normalised basal LC3‐vesicle levels in *ATG4D*‐mutant dog cells but decreased LC3 flux below the level observed in untreated WT cells. Testing lower concentrations of these drugs might be informative. Moreover, testing aciclovir homologues with either side‐chain or main‐chain modifications, prodrugs with improved cellular penetration or salt forms exhibiting higher water solubility compared with aciclovir might be rewarding with respect to deriving structure–activity relationships of guanosine analogues for efficient interference with basal autophagy [46]. In addition, they may help to clarify the mechanism of how guanosine analogues interfere with basal autophagy without affecting induced autophagy.

In addition to NVSD, other promising cellular targets relying on basal autophagy include cancer stem cells and precursor cells of EBV carcinoma [47, 48]. Interestingly, aciclovir in combination with IFNγ or valganciclovir plus bevacizumab has been implicated in glioblastoma treatment [49, 50]. Therefore, modulation of autophagy by guanosine analogues might be a promising strategy for the treatment of additional diseases.

### Anisomycin

Anisomycin is a protein synthesis‐inhibiting antibiotic isolated from *Streptomyces griseolus* and a potent activator of mitogen‐activated protein kinases (MAPK) such as JNK and p38. Our analysis identified anisomycin as a potent inhibitor, decreasing GFP‐LC3‐positive vesicles and both GFP‐LC3 and p62 flux in starved A549 cells. Notably, in A549 cells, the effect was strongly concentration‐dependent, with the strongest inhibition of autophagy observed at the lowest concentrations. Anisomycin also decreased autophagosome accumulation and autophagy flux in *ATG4D*‐mutant dog cells. Of note, anisomycin was shown to activate p38 MAPK, which subsequently binds p38IP and translocates to the nucleus [34]. In turn, nuclear p38IP is no longer available for the trafficking of Atg9, which is required for starvation‐induced autophagy [34]. Phosphorylation of Atg5 by Gadd45β‐induced p38 MAPK might be an alternative pathway of p38 MAPK inhibiting autophagy [51]. However, the autophagy‐inhibiting activity of anisomycin increased with decreasing concentrations of the compound. Since decreasing anisomycin concentration should result in less p38 MAPK activation and anisomycin inhibited not only starvation‐induced but also basal autophagy in A549 cells, anisomycin may exert additional inhibitory effects on the autophagy pathway. Further work is required to clarify the molecular mechanism of anisomycin affecting autophagy. In addition, we provide evidence that anisomycin very effectively impairs the replication of RNA viruses such as IAV and SARS‐CoV‐2. Interestingly, autophagy‐modulating compounds were shown earlier to act as antivirals in SARS‐CoV‐2 infection [52].

### Vioprolides

Vioprolides C and D inhibited GFP‐LC3 flux in both fed and starved A549 cells, and p62 flux in starved A549 cells. Vioprolides C and D also inhibited LC3 flux in fed WT LR fibroblasts, while only vioprolide D inhibited the flux in starved WT LR fibroblasts. Vioprolides are cyclic peptides that have been identified in myxobacteria, soil‐living microorganisms with a rich secondary metabolism [44]. Vioprolide A targets NOP14, an essential factor for ribosome biogenesis, thereby inhibiting translation [33]. Furthermore, immunomodulatory activities have been described for vioprolide A. On the one hand, vioprolide A has anti‐inflammatory effects by inhibiting the translation of short‐lived proteins involved in NF‐κB activation, such as TNFR1, IκBα and importin‐α1 [53]. The latter is crucial for the nuclear translocation of the NF‐κB subunit p65/RelA, and thereby vioprolide A reduces p65/ReIA nuclear localisation and suppresses NF‐κB‐mediated gene activation. Importantly, the inhibition of NF‐κB activation by vioprolide A is NOP14‐dependent [53]. On the other hand, vioprolides do not induce the release of the pro‐inflammatory cytokine IL‐1β via the classical caspase‐1‐dependent inflammasome but via a non‐canonical caspase‐8‐dependent mechanism [54]. Vioprolide A inhibits the expression of the short‐lived anti‐apoptotic Bcl2 family proteins, resulting in Bax/Bak‐dependent intrinsic apoptosis, which subsequently activated caspase‐8 [54]. Thus, vioprolides may have opposing functions depending on the cellular context and the proteome they alter by translation inhibition. Regarding the impact of vioprolides on autophagy, no direct connection between NOP14 and autophagy has been described in the literature. However, the expression of key autophagy proteins such as LC3, ATG4 and ATG5 is induced upon autophagy induction [55]. Therefore, vioprolides may exert their effect on autophagy via translation inhibition. However, a direct effect on the autophagy machinery cannot be excluded.

### Autophagy modulation in LR dog fibroblasts

As reported earlier [14], ATG4D^A430T/A430T^ mutant LR fibroblasts showed higher levels of LC3‐positive vesicles and moderately elevated autophagic flux under basal conditions. Serum and amino acid starvation did not increase these levels further, unlike in WT fibroblasts. Furthermore, autophagy‐independent but LC3‐associated pathways, such as LC3‐dependent extracellular vesicle loading and secretion (LDELS), may be altered in ATG4D^A430T/A430T^ cells [56]. ATG4D mutant LR fibroblasts release increased amounts of extracellular vesicles under basal conditions but not starvation [14], which suggests a possible alteration in the LDELS pathway under basal conditions. Therefore, the increased levels of LC3‐positive vesicles could be related to the increased release of extracellular vesicles (under basal conditions), in addition to an increase in LC3‐positive autophagosomes.

ATG4D is thought to be the main ATG8 delipidating enzyme in mammalian cells [9]. In addition to delipidating ATG8 proteins during canonical macroautophagy, ATG4D (similar to ATG4B) also delipidates ATG8 proteins from phosphatidylserine in a process called conjugation of ATG8 to single membranes (CASM), which can be active during phagocytosis, macropinocytosis, entosis and LC3‐associated endocytosis [57]. Vacuoles that accumulate in the pancreatic acinar cells and dorsal root ganglion neurons of the NVSD dogs were shown to be positive for the lysosomal membrane protein LAMP2, ATG4D, LC3 and transferrin receptor (a marker of the endocytic recycling pathway) [14]. It is possible that these vacuoles arise due to disturbed delipidation during either autophagy or CASM. However, vacuolisation was not observed in fibroblasts of the affected dogs, and we did not observe vacuolisation in the ATG4D^A430T/A430T^ mutant fibroblasts used in this study.

Decrease in LC3 delipidation could affect the amount of LC3‐positive vesicles in the ATG4D^A430T/A430T^ mutant fibroblasts. However, the A430T mutation is located outside the endopeptidase domain of ATG4D [58], suggesting the mutant enzyme likely retains enzymatic activity. Alanine 430 is located in the C‐terminal BH3 domain, upstream of the BH3 motif located at amino acids 453–460 [13, 59]. Furthermore, changes in delipidation activity of the mutant ATG4D, if any, would be expected to change the amount of LC3 vesicles under both basal and starvation conditions, and disturbed delipidation of ATG8 is expected to decrease autophagic flux. Because we observed elevated LC3 vesicle levels and increased autophagic flux only under basal conditions, our findings support the notion that the elevation was not due to decreased delipidation activity of the mutant ATG4D^A430T/A430T^. Of note, the C‐terminal domain of ATG4D is well conserved between dog and human proteins, and a frameshift mutation at amino acid 437 causes a neurodevelopmental disease in humans [12]. This frameshift also deletes the C‐terminal LIR motif of ATG4D.

At present, it is unknown whether the NVSD disease pathogenesis is related to alterations in autophagy, LDELS, CASM or other unknown processes. In future research, the effects of the drugs could be tested for the treatment of NVSD using neuronal cell cultures or animal models.

## Conclusion and limitations of the study

Here, we described a screening method employing high‐content microscopy to investigate the effect of commercial and natural substances on basal and induced autophagy. While the formation of GFP‐LC3‐ and endogenous LC3‐positive vesicles is widely used as an indicator of autophagosome accumulation, LC3 is not exclusively localised to autophagic structures (including phagophores, autophagosomes and amphisomes). It can also associate with other cellular structures, including those involved in LDELS [56] and LC3‐associated phagocytosis [60]. Thus, increases in GFP‐LC3‐ or LC3‐positive vesicles may reflect changes in other processes in addition to autophagy. However, we also used p62 as an additional autophagy marker.

We identified re‐purposing possibilities for compounds with known activities as well as novel activities of less‐well‐characterised natural compounds. The effect observed for anisomycin on SARS‐CoV‐2 replication is striking. However, although a connection between autophagy and coronavirus replication has been made in the past, we cannot state for certain whether anisomycin affects SARS‐CoV‐2 replication via autophagy modulation. Moreover, using LR cells harbouring an ATG4D mutation, which is associated with NVSD, we pinpoint a potential medical application of the identified compounds. However, the exact cellular mechanism of action of all the compounds identified here will have to be elucidated in future studies.

This study used cultured immortalised cell lines and fibroblasts isolated from tissue biopsies, which do not fully recapitulate the complex cellular environment found *in vivo*. While the cellular models are valuable for understanding basic processes, they lack stromal and extracellular matrix components and immune cells that can influence cellular behaviour. Genetic and epigenetic variations between cell lines and primary tissues can lead to differences in response to treatments. These issues affect the accuracy of transferring the findings to a clinical setting. Therefore, while our results provide important insights, further validation in more physiologically relevant models, including primary cells, organoids and animal models, is necessary to further assess the clinical relevance of our findings.

## Materials and methods

### Reagents

Aciclovir and famciclovir were purchased from Hölzel Diagnostika and TargetMol, respectively. Anisomycin (A9789) was obtained from (Sigma‐Aldrich, Steinheim, Germany). Bafilomycin A1 (ALX‐380‐063‐M001), chloroquine (51005) and rapamycin (51031) were purchased from Enzo (Lörrach, Germany). Vioprolide C and vioprolide D were produced as described earlier [44].

### Cell culture

A549 (RRID: CVCL_0023) and A549 GFP‐LC3 [41] cells were cultured in Ham's F‐12K Nutrient Mix supplemented with 10% foetal calf serum (FCS) and 1% penicillin–streptomycin at +37 °C and 5% CO_2_. Madin‐Darby canine kidney cells (MDCK, ATCC CCL‐34, RRID: CVCL_0422), which are readily infectable and often used in IAV research, were maintained in Dulbecco's modified Eagle's medium (DMEM) supplemented with 10% FCS and 2 mm l‐glutamine at +37 °C in 5% CO_2_. VeroE6 (ATCC CRL‐1586, RRID: CVCL_0574), which originate from African green monkey kidney and are commonly used in Coronavirus research and Caco‐2 (RRID: CVCL_0025) cells were cultured in DMEM supplemented with 10% (v/v) FCS, 1% (v/v) non‐essential amino acids, 100 IU·mL^−1^ penicillin, 100 μg·mL^−1^ streptomycin and 2 mm l‐glutamine. Fibroblasts from Lagotto Romagnolo (LR) dogs carrying a mutation in the ATG4D gene (c.1288G>A; p.Ala430Thr) and fibroblasts from healthy controls were grown in MEMalpha medium (L0476; Biowest, Nuaillé, France) supplemented with 5% FCS (S1810; BioWest, Nuaillé, France). Dog cells were established (permit: ESAVI/6054/04.10.03/2012) and kindly provided by Dr Pernilla Syrja [14]. No additional dogs were sacrificed for the current study. Because mycoplasma contamination can impact autophagic flux, all cell cultures were tested regularly to be mycoplasma‐free using Venor®GeM OneStep kit (11‐8050; Minerva Biolabs, Berlin, Germany).

### Bacterial strain and culture conditions

*Staphylococcus aureus* strain SH1000‐GFP was grown in tryptic soy broth (TSB) (22092; Sigma‐Aldrich), supplemented with 30 μg·mL^−1^ chloramphenicol (3886.2; Carl Roth, Karlsruhe, Germany). For growth on solid medium, 1.5% (w/v) agar (214010; BD Biosciences, Heidelberg, Germany) was added. A bacterial overnight culture was diluted to an optical density at 600 nm (OD_600_) of 0.05, further grown with agitation at +37 °C to an OD_600_ of 1; subsequently, it was used for infection of eukaryotic cells.

### Compound screening

Cells were seeded on 96‐well plates at a density of 3.5 × 10^4^ cells per well in 100 μL of their respective medium. The plates were left at room temperature for 1 h without moving them to guarantee an even distribution of the cells on the well bottom and then cultured overnight at +37 °C and 5% CO_2_. On the next day, compounds were added at a concentration of 10 μm in either 100 μL of the normal medium or 100 μL of Hank's balanced salt solution (HBSS) to induce starvation conditions and then cultured at +37 °C and 5% CO_2_ for 3 or 1.5 h, respectively. Subsequently, cells were carefully washed once with phosphate‐buffered saline (PBS), fixed in 4% formaldehyde for 20 min at room temperature and washed again twice with PBS. Nuclear staining was performed using 0.05% Hoechst 33342 solution in PBS for 10 min at room temperature. After another washing in PBS twice, cells were stored in PBS at +4 °C and were ready for measurement with the automatic fluorescence microscope (ImageXpress Micro XLS Widefield High‐Content Imaging system; Molecular Devices, Munich, Germany). For each well, pictures at nine different sites were taken using exposure times of 150 ms for Hoechst 33342 and 1000 ms for GFP. Analysis was performed using the metaxpress software (Molecular Devices) with the following settings: vesicle width 0.5–5 μm and necessary intensity 2000 grey levels above the local background as determined from the GFP channel. The size of nuclei was 5–30 μm and the necessary intensity was 2000 grey levels above the local background as determined from the DAPI channel. The amount of GFP‐LC3 vesicles was expressed as the total area of GFP‐LC3 puncta per cell and normalised to the untreated cells. Cell viability upon compound treatment was analysed by counting Hoechst 33342‐stained nuclei.

Compounds were added to cells in complete medium (fed condition) as well as amino acid‐ and serum‐free medium (starved condition) and the relative changes in vesicular LC3 were calculated in comparison with the respective negative control (fed treated versus non‐treated and starved treated versus non‐treated, respectively). Two independent screening experiments were performed. The compounds that increased or decreased the amount of vesicular LC3 by more than a range of three standard deviations around the negative control were classified as hits.

### Cell viability assays

Cell viability was analysed by resazurin assay. Resazurin sodium salt (20 μg·mL^−1^; Sigma‐Aldrich) is reduced by mitochondrial enzymes to fluorescent resorufin. Fluorescence was measured in a CLARIOstar Plus plate reader (BMG Labtech, Ortenberg, Germany) with an excitation wavelength of 540 nm, an emission of 590 nm and a cut‐off of 590 nm. For SARS‐CoV‐2 infection, viability was monitored by thiazolyl blue tetrazolium bromide (MTT) assay (Sigma‐Aldrich).

### Immunocytochemistry and fluorescence microscopy

A549 cells expressing GFP‐LC3 were grown on glass coverslips and fixed with 4% paraformaldehyde (PFA) in PBS (137 mm NaCl, 2.7 mm KCl, 10 mm Na_2_HPO_4_, 1.8 mm KH_2_PO_4_, pH 7.4) at room temperature for 20 min. Permeabilisation was carried out with 0.2% saponin (47036; Sigma‐Aldrich) in PBS for 10 min and blocking with 3% bovine serum albumin and 0.2% saponin in PBS for 30 min. Cells were stained with mouse anti‐p62 (BD610832; BD Biosciences) or mouse anti‐LAMP2 (H4B4; Developmental Studies Hybridoma Bank), followed by anti‐mouse IgG Alexa Fluor 488 (AF11001; Thermo Fisher, Waltham, MA, USA). Coverslips were then mounted on microscope glass slides with Mowiol (475904; Calbiochem, Darmstadt, Germany) containing the antifading agent 1,5‐Diazabicyclo [2.2.2] octane (DABCO, D‐2522; Sigma) and the nuclear staining agent 4′,6‐diamidine‐2phenyl indole (DAPI, D1306; Invitrogen, Carlsbad, CA, USA).

LR dog fibroblasts were stained with mouse anti‐LC3 (CTB‐LC3‐2‐IC; Cosmo Bio, Carlsbad) followed by anti‐mouse IgG Alexa Fluor 488 (AF11001; Thermo Fisher).

Images were acquired with a Marianas CSU‐W1 confocal microscope with a 63× objective (3i). At least 40 cells from two biological replicates were analysed per sample. The area and intensity of GFP‐LC3, endogenous LC3 and endogenous p62 puncta were determined using fiji [61]. Individual cells were segmented; the background was subtracted globally, and the positive puncta on all optical sections were segmented. The total area and total intensity of the segmented puncta were measured and summed up for each individual cell. The values presented in the figures were calculated by multiplying the total area of the puncta with the total intensity of puncta and by normalising this to the cell area. Autophagic flux was calculated by subtracting the GFP‐LC3, LC3 or p62 values, determined by the method explained above, of samples cultured without bafilomycin A1 from the values of the corresponding samples treated with bafilomycin A1.

Co‐localisation of GFP‐LC3 and LAMP2 was determined using fiji. Individual cells were segmented, and the background was subtracted globally in the LC3 and LAMP2 channels, respectively. LC3‐positive puncta were segmented in all optical sections. Finally, the Coloc 2 plugin was used to determine the Mander's coefficient for the proportion of LC3 signal positive for LAMP2 in the segmented areas.

### *S. aureus* infection and infection kinetics

For *S. aureus* infection, 2.5 × 10^4^ A549 cells per well were seeded in a black 96‐well optical bottom tissue culture plate (P8991; Sigma‐Aldrich). After 24 h, the culture medium was exchanged for 100 μL fresh medium. Afterwards, cells were infected with *S. aureus* SH1000‐GFP early stationary phase cultures at an MOI of 4 for 90 min. Subsequently, cells were treated with 10 μg·mL^−1^ lysostaphin (L9043; Sigma‐Aldrich) for 15 min to kill all extracellular bacteria (time point 0 h). The medium was exchanged by fresh medium supplemented with 0.05% Hoechst 33342 to stain the nuclear DNA of the cells. Either 1 μL of compound (1 mm) or 1 μL DMSO as solvent control was added to the wells. The plate was transferred to the incubation chamber (+37 °C) of the automatic microscope (Cytation5; Agilent BioTek, Santa Clara, CA, USA). Pictures were taken every hour for a total of 12 h (objective: 20×; channel 1: DAPI 377477; channel 2: GFP 469525).

### *S. aureus* growth curves

Growth curve assessments were performed in 96‐well plates. One hundred microlitres of TSB/well was inoculated with a 1 : 100 dilution of an overnight culture of *S. aureus* SH1000‐GFP. Either 1 μL of compound (1 mm) or 1 μL of DMSO was added per well. The plate was placed into the incubation chamber (+37 °C) of the Cytation5 and incubated for 210 min with shaking. OD_600_ and GFP fluorescence were measured every 10 min.

### Influenza a virus infection and neuraminidase activity

Influenza A/Puerto Rico PR/8/1934 (IAV, H_1_N_1_, Mount Sinai Strain) was kindly provided by Stephan Ludwig (Virology, ZMBF, Muenster, Germany). Infections of MDCK cells with IAV were performed at an MOI indicated in the figure legend [62]. Sixteen hours after infection, test compounds were added at the concentrations indicated in the figure legend. To determine the amount of infectious IAV, nearly confluent monolayers of A549 cells grown in F12K medium were infected with IAV at an MOI of 2 for 1 h in PBS containing 0.5% bovine serum albumin (BSA) and 1 μg·mL^−1^ acetylated trypsin (Sigma). Fresh medium (F12K plus 1% BSA) containing the test compounds was then added for 16 h at +37 °C. Subsequently, supernatants were harvested on ice, passed through 0.2 μm membrane filter units (Merck Millipore, Burlington, MA, USA) and stored in aliquots at −70 °C for later analysis. Neuraminidase (NA) activity in the IAV‐containing supernatants was detected by the NA‐Star XTD® Influenza Neuraminidase Inhibitor Resistance Detection Kit (Applied Biosystems, Waltham, MA, USA). Briefly, to perform the assay, 25 μL of twofold dilution of compound‐treated cell culture supernatant in NA‐Assay Buffer were incubated for 10–30 min with 10 μL NA‐Star XTD substrate in a white 96‐well assay plate (Berthold 96 No: 23302). The plates were placed in a luminometer (Berthold TriStar2), and 60 μL of the accelerator solution (NA‐XTD) was added to each well. Glow‐type luminescence was determined within 1 h using a 0.5–1 s per well read. In case the former NA Star substrate was used, the accelerator was injected using the onboard injector of Berthold TriStar2, which triggered immediate light emission from the cleaved NA‐Star substrate (flash type assay). Light signal intensity was measured within seconds of accelerator injection using a 0.5–1 s per well read. The entire assays were completed in less than 1.5 h. To avoid the effects of medium constituents, values achieved from at least fourfold diluted viral supernatants were used for calculation.

### SARS‐CoV‐2 infection assay

To determine the antiviral activity of anisomycin, VeroE6 or Caco‐2 cells were seeded at a density of 8 × 10^4^ cells per well in a 24‐well plate. After the cells were attached to the plate, they were treated with anisomycin in a dose‐dependent manner for 1 h and subsequently infected with hCoV‐19/Germany/BY‐Bochum‐1/2020 (B.1.1.70; GISAID accession ID: EPI_ISL_1118929; [63], MOI 1) for 1 h. The cells were washed thrice with PBS and again treated with the drug as indicated. One day postinfection, the supernatant was collected, and infectious viral titres were determined by an end‐point dilution assay on VeroE6 cells seeded at 1 × 10^4^ cells per well in a 96‐well plate to calculate the 50% tissue culture infectious dose (TCID_50_)·mL^−1^, which quantifies virus titres by end‐point dilution via the cytopathic effect induced by the virus. Additionally, infected cells were harvested, and RNA was isolated using the RNeasy Kit (Qiagen, Hilden, Germany) according to the manufacturer's instructions. Cell viability was monitored by thiazolyl blue tetrazolium bromide.

### Time‐of‐addition kinetics

VeroE6 cells were seeded at 8 × 10^4^ cells per well in a 24‐well plate. Cells were then treated with 0.1 μm anisomycin for different time periods starting from 16 h preinfection to 8 h postinfection. The cells were infected with hCoV‐19/Germany/BY‐Bochum‐1/2020 (B.1.1.70; GISAID accession ID: EPI_ISL_1118929; [63], MOI 1) for 1 h. The cells were then washed thrice with PBS and again treated with the drug as indicated. One day postinfection, the supernatant was collected, and infectious viral titres were determined by an end‐point dilution assay on VeroE6 cells.

### RT‐qPCR

RNA was isolated from cell lysates using the RNeasy Kit (Qiagen) according to the manufacturer's instructions. Subsequently, RNA was reverse transcribed into cDNA using a PrimeScript First Strand cDNA Synthesis Kit (Takara Bio, Saint‐Germain‐en‐Laye, France). Viral RNA from the cell culture supernatant was isolated using the RNA Blood Kit (Qiagen). To determine relative SARS‐CoV‐2M‐gene expression, gene‐specific primers and probes [64] and GoTaq Probe qPCR Master Mix (Promega, Madison, WI, USA) were used according to the manufacturer's instructions. RT‐qPCR was performed on a LightCycler 480 II (Roche, Mannheim, Germany) instrument.

### Immunofluorescence staining for SARS‐CoV‐2 infections

Infected and control cells were stained as described recently [65]. In brief, the cells were fixed with 4% PFA at +4 °C overnight. Subsequently, the cells were permeabilised with 0.2% Triton X‐100 (Carl Roth) for 5 min and blocked in 5% horse serum (Gibco) for 1 h. The cells were then stained overnight with agitation for the presence of dsRNA (anti‐dsRNA; Sigma, MABE1134 diluted 1 : 1000 in 5% horse serum). The secondary antibody (donkey anti‐mouse IgG Alexa Fluor 555; Invitrogen, SAB4600060 diluted 1 : 1000 in 5% horse serum) was incubated for 2 h. Hereafter, the nuclei were stained with DAPI (Invitrogen, diluted 1 : 10 000 in H_2_O) for 5 min. In between each step, the cells were washed thrice with PBS. Imaging was performed with the Keyence BZ800 microscope.

### Statistics

Screening data were analysed using graphpad prism version 5.04 (GraphPad Software, La Jolla, CA, USA). To detect toxic effects, samples with nuclear counts reduced by 30% compared with the fed control cells, or the median of all cells in the case of the high‐throughput screening of test libraries, were excluded from the analysis. The treatment was assessed as being toxic if a reduction was observed in independent experiments. For assessment of the quality of our high‐content screening system, the *Z*′‐factor, known for statistical evaluation and validation of high‐throughput screening assays, was calculated [66]. For the high‐throughput screening of test libraries, all samples with a GFP‐LC3B‐positive vesicle area below or above a range of three times the standard deviation of the fed control cells were classified as hits, and the applied compounds were considered potential autophagy modulators if similar results were obtained in independent tests. This range was chosen because, for approximately normal data sets, the values within three standard deviations above or below the mean account for about 99.7% of the set. Following the three‐sigma rule of thumb, it is empirically useful to treat this 99.7% probability as near certainty and assume that nearly all values lie within three standard deviations of the mean [67]. Replicate scores per treatment for the controls or compound titration experiments were summarised using the mean, and statistical significance was calculated with an unpaired two‐sided Student's *t*‐test. For the confocal microscopy of GFP‐LC3‐expressing A549 cells and LR fibroblasts, statistical analyses were performed by the non‐parametric Kruskal–Wallis test and *post hoc* Dunn's multiple comparisons test, using the graphpad prism software (version 10.1.2).

## Conflict of interest

The authors declare no conflict of interest.

## Author contributions

JF, YYB, HGMR, SM, TLM, SP, MW, E‐LE and IS planned the experiments. JF, YYB, HGMR, SM and TLM performed the experiments. JF, YYB, HGMR, SM, TLM, SP, MW, E‐LE and IS analysed the data. FS, JH, SP, UB, MB and RM contributed the essential reagents and helped with the screening procedure. MW, E‐LE and IS supervised the study. JF, YYB, HGMR, SM, E‐LE and IS wrote the paper. All authors read and approved the final version of the manuscript.

## Data Availability

The data that support the findings of this study are available from the corresponding author upon reasonable request. Data are located in controlled access data storage at the Ruhr University Bochum.
